# Spatial transcriptomics uncovers immune-cell plasticity and dedifferentiation signatures in aggressive lung adenocarcinoma subtypes

**DOI:** 10.3389/fimmu.2025.1620886

**Published:** 2025-08-21

**Authors:** Huiyan Deng, Qingyi Liu, Ziqiang Tian, Guangjie Liu, Shize Wang, Di Liang, Yun Wang, Yaqing Han, Shaonan Xie

**Affiliations:** ^1^ Department of Pathology, The Fourth Hospital of Hebei Medical University, Shijiazhuang, Hebei, China; ^2^ Department of Thoracic Surgery, The Fourth Hospital of Hebei Medical University, Shijiazhuang, Hebei, China; ^3^ Cancer Institute in Hebei Province, The Fourth Hospital of Hebei Medical University, Shijiazhuang, Hebei, China; ^4^ Department of Pulmonary Medicine, The Fourth Hospital of Hebei Medical University, Shijiazhuang, Hebei, China

**Keywords:** lung adenocarcinoma, heterogeneity, spatial transcriptomics, bioinformatics, molecular characteristics of subtype, biological processes in tumor development

## Abstract

Intrinsic genetic alterations and dynamic transcriptional changes contribute to the heterogeneity of solid tumors. Lung adenocarcinoma (LUAD) is characterized by its significant histological, cellular and molecular heterogeneity. The present study aimed to study the spatial transcriptomics of primary LUAD with initial hopes to decipher molecular characteristics of subtype transitions in LUAD progression, offering new insights for novel therapeutic strategies. Spatial transcriptomics libraries were first generated from tumor samples collected from patients with LUAD who underwent surgical resection in The Fourth Hospital of Hebei Medical University in 2022 and were sequenced using Illumina NovaSeq 6000 system. The processed data were analyzed for differential gene expressions and networks, and were annotated according to cell type, spatial ligand-receptor interaction and trajectory inference. Our analysis revealed 34 annotated cell types, with cancer-associated fibroblasts (CAFs) being the most abundant, playing a crucial role in tumor microenvironment remodeling and prognosis. We noted significant spatial correlations between various immune cells and found that different histological subtypes displayed unique cell composition profiles, particularly in the micropapillary subtype, which exhibited higher macrophage proportions and distinct gene expression pathways related to extracellular matrix organization and receptor tyrosine kinase signaling. Additionally, we explored the dedifferentiation states within these subtypes, identifying that region with higher dedifferentiation scores corresponded to increased tumor invasiveness and potential drug resistance. Our findings demonstrate dynamic biological changes and dedifferentiation states of tumor subtypes during the progression process. This study reveals important biological processes in tumor development and may offer valuable guidance for future therapeutic strategies.

## Introduction

The development of cancer is driven by the accumulation of changes that affect the structure and function of the genome (1). Changes in the morphology, function, and behavior of tumor cells to adapt, known as cellular plasticity, are important features of tumor progression and metastasis and are associated with a de-differentiated state related to treatment resistance and poor clinical outcomes (2). Both intrinsic and extrinsic factors, such as genetic variations, epigenetic modifications, transcriptional changes, and treatment-induced selective pressure, shape the plasticity of cancer cells, thereby promoting heterogeneity both between and within tumors (3). Cell fate transitions, including epithelial-mesenchymal transition (EMT), mesenchymal-epithelial transition (MET), and cancer stem cell formation, are fundamental processes in cellular reprogramming and tumor metastasis (4, 5). Cellular plasticity is often associated with a low-differentiated phenotype of genes, which can be mediated by transcription factors and microRNAs that regulate the tumor microenvironment, cell polarity, adhesion, and motility (6).

Lung adenocarcinoma (LUAD), one of the most common and lethal malignant tumors, is characterized by significant histological heterogeneity, as well as cellular and molecular heterogeneity (7). Multiple histological subtypes may coexist within the tumor tissue. Some high-grade subtypes (such as micropapillary, solid, and complex glandular structures) exhibit highly aggressive behavior and are associated with poor prognosis, while low-grade subtypes (such as lepidic and acinar) display relatively slow growth characteristics (8). Although extensive genetic and epigenetic heterogeneity has been demonstrated in LUAD, the molecular characteristics and biological interactions of different histological subtype transitions within LUAD tissues remain unclear (9).

Single-cell sequencing technology has advanced the in-depth study of intratumoral heterogeneity, tumor microenvironment, progression and metastasis mechanisms of lung adenocarcinoma, as well as resistance mechanisms, at the single-cell resolution level (9–14). For example, comprehensive characterization of tumor-infiltrating lymphocytes has revealed dynamic changes in T cell functional states in non-small cell lung cancer (15). Additionally, single-cell transcriptomic analysis of *in situ* adenocarcinoma, microinvasive adenocarcinoma, and invasive adenocarcinoma depicts the dynamic evolution from *in situ* adenocarcinoma to invasive adenocarcinoma (16). Single-cell RNA sequencing (scRNA-seq) identifies cell subpopulations within tissue but does not capture their spatial distribution nor reveal local networks of intercellular communication acting *in situ* (17). Spatial transcriptomics sequencing can preserve spatial information within tissue samples while measuring gene expression, allowing for precise localization of the expression of different genes in specific areas of the tissue. It enables the observation of interactions between different cell types and their distribution within the tissue, thereby complementing the limitations of single-cell transcriptomics sequencing (18).

**Figure 1:**
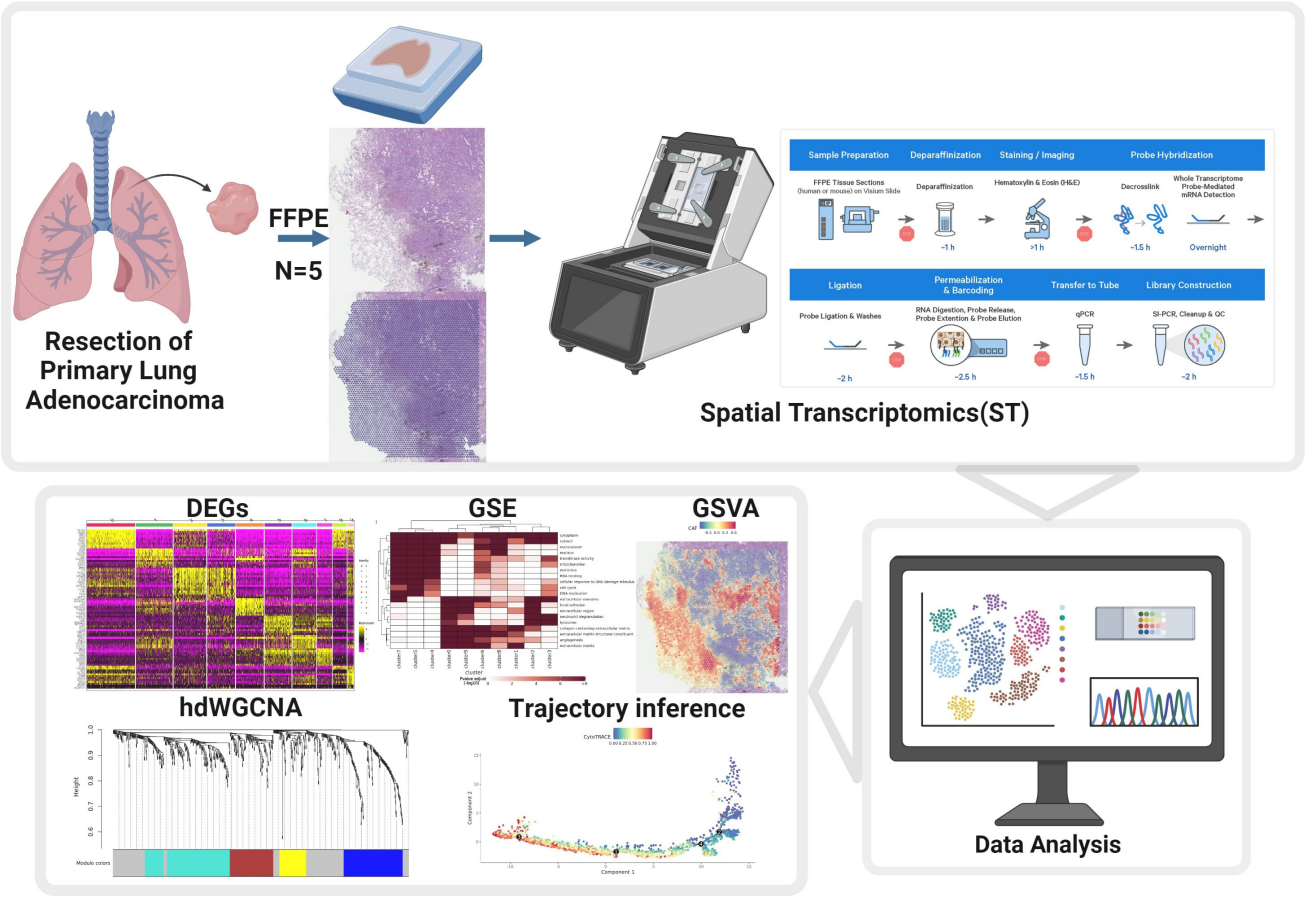
Schematic overview of the study design. Workflow illustrating the collection of FFPE primary lung adenocarcinoma samples, preparation for spatial transcriptomics (ST), data acquisition using the 10x Visium platform, and downstream bioinformatics analysis including identification of differentially expressed genes (DEGs), hdWGCNA, gene set enrichment (GSE), gene set variation analysis (GSVA), trajectory inference, and data analysis.

The present study applied spatial transcriptomics technology to primary LUAD containing high-grade histological subtypes to explore the spatial cellular composition differences between different histological subtypes, the molecular characteristics driving these subtypes, and the differences in the tumor microenvironment. Overall, these results would provide molecular characteristics of subtype transitions in LUAD progression, offering new insights for novel therapeutic strategies (**Figure 1**).

## Materials and methods

### Human specimens

Specimens were collected from patients with LUAD who underwent surgical resection at the Fourth Hospital of Hebei Medical University in 2022. Among the 188 patients with a micropapillary histological pattern, we selected five cases where the micropapillary pattern constituted between 30% and 50%. Two experienced pathologists reviewed each slide to identify suitable tissue sections for spatial transcriptomics analysis and annotated the tumor slides for different histological subtypes, including lepidic (LEP), acinar (ACI), micropapillary (MIP) subtypes, and normal tissue (N). The pathological diagnoses for each slide were based on the 2021 World Health Organization (WHO) classification of LUAD (19) and the new grading system proposed by the International Association for the Study of Lung Cancer Pathology Committee (20). This study was conducted in accordance with the Declaration of Helsinki (2013 revised version) and was approved by the Ethics Committee of the Fourth Hospital of Hebei Medical University (Institutional Review Board number 2021Ky103). All patients signed written informed consent prior to participating in this study.

### Sample preparation

The RNA quality of FFPE tissue blocks was assessed by calculating the DV200 of RNA extracted from FFPE tissue sections, following the Qiagen RNeasy FFPE Kit protocol. Five-micrometer sections were placed on Sigma-Aldrich Poly Prep Slides in accordance with the Visium CytAssist Spatial Gene Expression Protocols for FFPE Tissue Preparation Guide (10x Genomics, CG000518 Rev C). After drying overnight, the slides were incubated at 60°C for 2 hours. Deparaffinization was performed according to the Visium CytAssist Spatial Gene Expression for FFPE — Deparaffinization, Decrosslinking, Immunofluorescence Staining & Imaging Protocol (10x Genomics, CG000519 Rev B). The sections were then stained with hematoxylin and eosin and imaged at 20x magnification using the brightfield imaging settings on a Leica Aperio Versa8 whole-slide scanner. Decrosslinking of the H&E stained sections was conducted immediately afterward. Next, human whole transcriptome probe panels were added to the tissue. After the probe pairs hybridized to their target genes and ligated to one another, the slides were placed on the Visium CytAssist instrument for RNase treatment and permeabilization. The ligated probes were subsequently hybridized to the spatially barcoded oligonucleotides in the Capture Area. Spatial transcriptomics libraries were generated from the probes and sequenced on the Illumina NovaSeq 6000 system (conducted by Beijing Novogene Technology Co., Ltd.).

### ST data processing

Raw sequencing data were processed using Space Ranger pipelines (version 2.0.0), including tissue detection, fiducial detection, read alignment, barcode and UMI counting against reference genome GRCh38 (version 3.0.0, pre-built by 10x Genomics). Feature-spot matrices were generated based on spatial barcodes, and then analyzed with the Seurat R package (V3.1.2) (21). To normalize sequencing depth variance across spatial spots, especially for technical artifacts and tissue anatomy, we used SCTransform function based on regularized negative binomial regression to normalize molecular count data, and detect high-variance features. Data from the five spatial slides were integrated using Seurat’s reciprocal principal component analysis (RPCA) integration workflow to correct for potential batch effects. This involves identifying anchors between datasets (slides) and using these anchors to harmonize the gene expression data across slides. The effectiveness of batch correction was confirmed by ensuring that spots did not primarily cluster by sample origin in the UMAP projections. Dimensionality reduction was performed with principal component analysis (PCA), then followed by a shared SNN construction based on Jaccard index between spots with the first 50 dimensions. Cluster determination was performed using the FindClusters function at resolution 0.6 by a SNN modularity optimization. The top 20 PCA dimensions were used for UMAP dimensional reduction. Subsequently, clusters in UMAP space were visualized by DimPlot and SpatialDimPlot functions. Spatially variable features that correlate with spatial subtypes were identified by FindSpatiallyVariables function with markvariogram method. To identify differentially expressed genes for each cluster, we used FindAllMarkers function in Seurat with default parameters, and genes with logFC > 0.25 and adjusted P value < 0.05 were considered as significantly different. The logFC threshold of 0.25 was chosen to capture a broader range of potentially relevant gene expression changes while maintaining statistical rigor in conjunction with the adjusted p-value cutoff.

### Enrichment analysis

For genes associated with differentiation, R package clusterProfiler (version v4.5.1) (22) was used to perform GO/KEGG/REACTOME enrichment analysis using corresponding gene sets. P value was adjusted for multiple comparisons by Benjamini-Hochberg correction. Significant thresholds were set to a q-value cutoff of 0.05.

### Cell-type annotations

To annotate cell types to spatial spots, we employed a multi-step approach. Initially, cell type deconvolution was performed using the SpaCET R package (23), which utilizes reference single-cell RNA sequencing datasets from human lung tissue to estimate the proportion of various cell types within each spatial spot. Each spot was then assigned a dominant cell type based on the highest estimated proportion. For further characterization of functional states and pathway enrichments within these annotated spots, we applied gene set variation analysis (GSVA) (24). Signature gene sets for specific cell types and functional states were derived from established literature and the SpaCET package. GSVA scores were calculated for each gene set per spot, allowing for the assessment of relative pathway activity within spatially defined regions and cell populations. This combined approach allowed for robust cell type identification and subsequent functional characterization.

### Spatial ligand-receptor interaction analysis

Spatial intra-celltype ligand-receptor interaction pairs were inferred using CellPhoneDB (version 3.1.0) (25) with a built-in database for humans. Metadata and count matrix files were used as input data, and other arguments were kept as default. P value was calculated using the proportion of means that exceeded the actual mean, and ranked based on its significance.

### Trajectory inference

To rank cells by their developmental potential, we used CytoTRACE (v.0.3.3). CytoTRACE is a statistical method, which uses transcriptional diversity as a proxy for developmental potential and assigns a CytoTRACE-score to each cell (26). CytoTRACE scores were calculated for each cluster independently, using default parameters. Cell cluster that has the lowest median CytoTRACE-score was used as the root for trajectory analysis. We utilized monocle2 (version 2.28.0) to generate pseudotime trajectories and identify differentially expressed genes between branches (27). Briefly, the highly variable genes (HVGs) identified by R package Seurat were selected as genes that define a cell’s progress. DDRTree algorithm was used to reduce data dimensionality, and cells were ordered along the trajectory according to pseudo times. To identify genes that were differentially expressed between the branches, a special statistical test named “branched expression analysis modeling” (BEAM) was used.

### Analysis of spatial gene expression programs

We performed high-dimensional weighted gene co-expression network analysis (hdWGCNA) (28) in our ST dataset. Significant DEGs identified between histologic sub-regions were used as input for hdWGCNA, and we applied the K-Nearest Neighbors algorithm to identify groups of similar spots by means of transcriptomics (termed metacells in this context, representing aggregated transcriptionally similar spots rather than individual cells) and constructed a metacell gene expression matrix. We obtained the module eigengene values, which describe the expression patterns of entire co-expression modules, and performed a differential module eigengenes analysis applying a Mann-Whitney U test. WGCNA was performed on each slide individually to account for sample-specific variations. For each slide, the soft power threshold was selected by analyzing the scale-free topology fit index, aiming for a signed R2 > 0.8 to ensure a scale-free network. Other parameters, such as minimum module size, were kept consistent across analyses to facilitate comparison.

## Results

### Spatial transcriptomic features of LUAD

**Figure 2:**
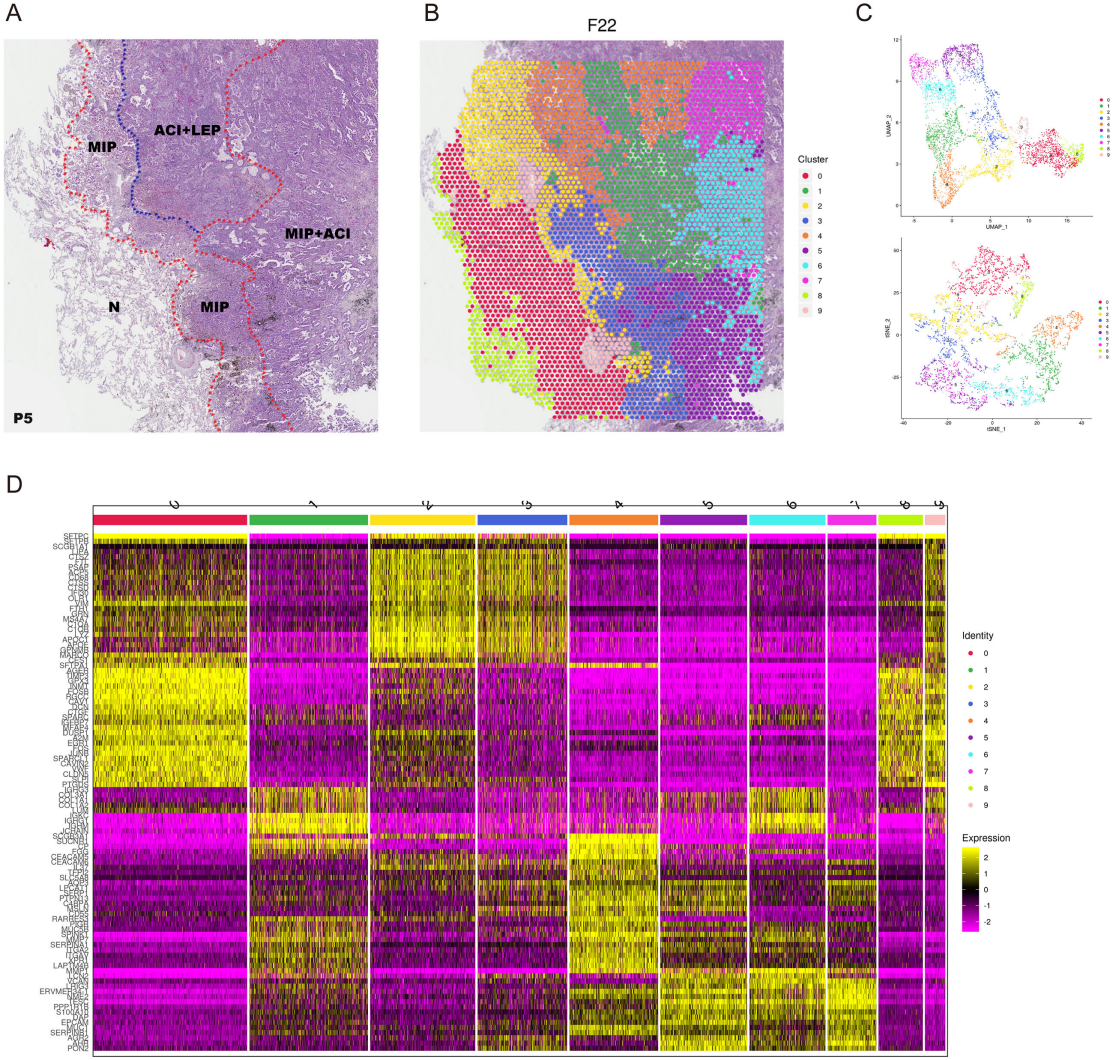
Spatial transcriptomic features of LUAD patient P5. **(A)** Hematoxylin and eosin (H&E) stained tissue section from patient P5 showing pathologist-annotated regions: Micropapillary (MIP), Acinar+Lepidic (ACI+LEP), MIP+ACI, and Normal (N). **(B)** Spatial map of P5 tissue spots colored by Seurat clusters. **(C)** UMAP visualization of spatial spots from P5, colored by Seurat clusters (top) and pathologist-annotated histological regions (bottom). **(D)** Heatmap showing expression of top differentially expressed genes across the 10 identified Seurat clusters in P5. Color bar indicates normalized gene expression levels.

LUAD can be classified into five histological patterns: lepidic, acinar, papillary, micropapillary, and solid. These patterns are factors influencing prognosis, particularly the micropapillary histological pattern, which is associated with poor outcomes. Most LUAD exhibit multiple histological patterns. To explore the differences in cellular composition and molecular subtypes among these patterns and to investigate the reasons for the poor prognosis in patients with the micropapillary pattern, we conducted spatial transcriptomics (ST) studies on samples from five patients who underwent radical resection and included the micropapillary histological pattern on the 10× Visium platform (**Supplementary Figure 1**, **Supplementary Table S1**). The heterogenous histology was as shown in **Figure 2A**. Transcriptomic data were obtained from a total of 21,617 spots, with a median number of genes captured per spot for each specimen being 6,914 (**Supplementary Figure 2**) and a median Unique Molecular Identifier of 21,855 (**Supplementary Table S2**). We performed clustering analysis (**Figure 2B**) and dimensionality reduction (**Figure 2C**) on the gene expression matrix using Seurat software. For example, the specimen from patient P5 included micropapillary (MIP), micropapillary mixed with acinar (MIP+ACI), acinar mixed with lepidic (ACI+LEP), and normal tissue (N), which could be categorized into 10 clusters based on differences in gene expression (**Figure 2D**) (**Supplementary Figure 3**) (**Supplementary Figure 4**, **Supplementary Table S3**).

**Figure 3:**
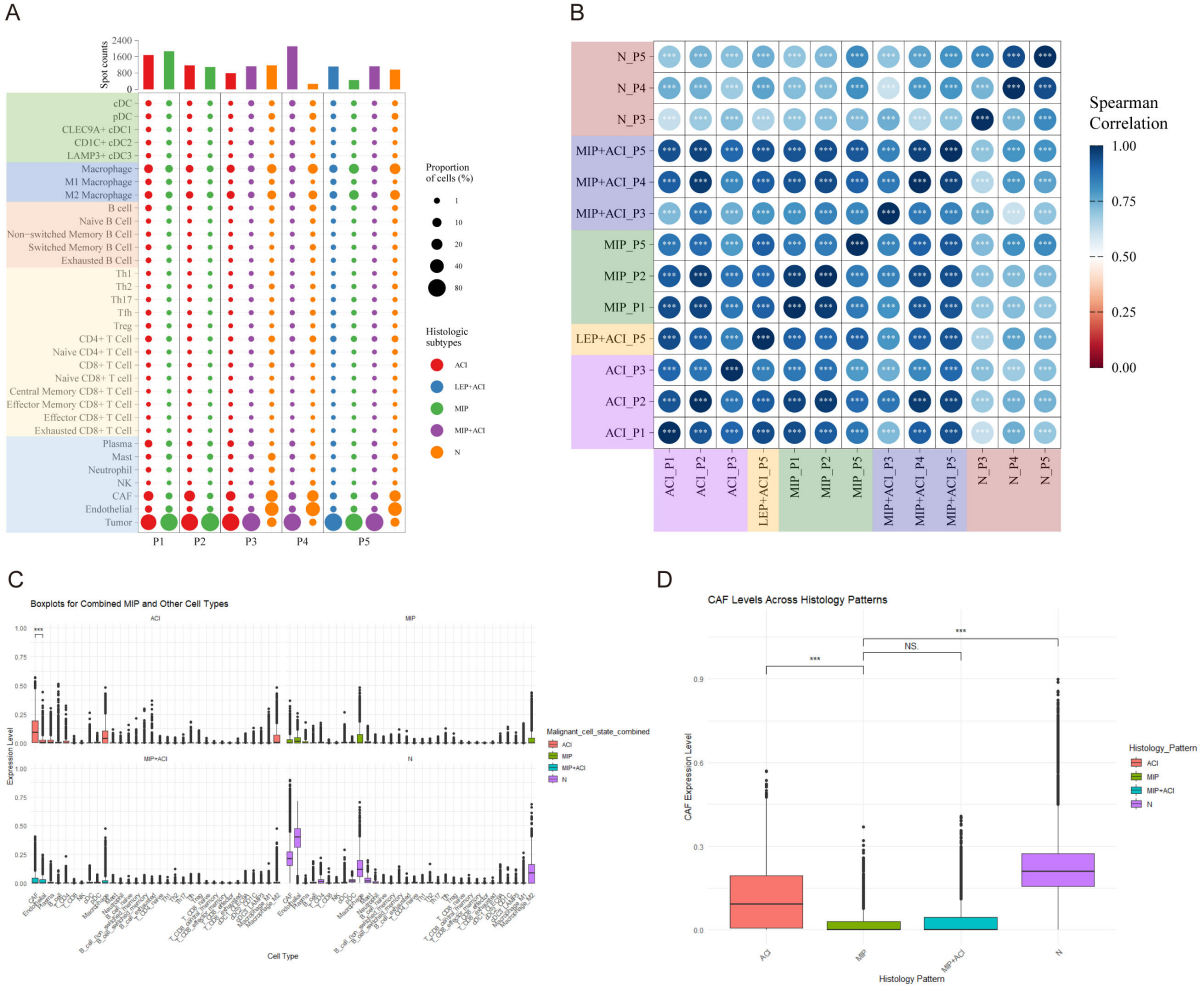
Cell type composition and correlation across LUAD histological subtypes. **(A)** Dot plot showing the proportion and number of spots for various annotated cell types across different histological subtypes (ACI, LEP, MIP, MIP+ACI, N) in patients P1-P5. Dot size indicates proportion of cells, and color indicates histological subtype. **(B)** Spearman correlation matrix of cell type compositions across different pathologist-annotated histological regions. Color intensity and circle size represent the correlation coefficient. ***p < 0.001. **(C)** Boxplots showing expression levels (arbitrary units based on deconvolution scores) of combined MIP and other cell types (top panel) and selected individual cell types (bottom panel) in ACI, MIP, and MIP+ACI regions. **(D)** Boxplot comparing Cancer-Associated Fibroblast (CAF) expression levels across ACI, MIP, MIP+ACI, and N histological patterns. ***p < 0.001, NS, not significant.

Based on the deconvolution results using the SpaCET package and reference single-cell datasets, we annotated the cell types in the spatial transcriptomics specimens. A total of 34 cell types were annotated, including tumor cells, cancer-associated fibroblasts (CAFs), and immune cells (macrophages, T cells, B cells, etc., along with their subgroups) (**Supplementary Figure 5A**; **Supplementary Table S4**). Overall, CAFs were found to be the most abundant cell type in LUAD (**Supplementary Figure 5B**). Macrophages, particularly M2 (M2-like) macrophages, were the second most abundant cell type. The cell annotation results for different histological patterns are shown in (**Figure 3A**). In samples P3-P5, a minor proportion of tumor cells was detected in regions annotated as “Normal” (N). These tumor cells were primarily located at the interface between the tumor mass and the adjacent normal tissue, likely representing microscopic infiltration or the inherent resolution limits of ST spots capturing signals from adjacent tumor areas (detailed mapping in **Supplementary Figure 7**). Using cell type correlation analysis, we observed significant spatial correlations between different cell types within the same spot, such as between T follicular helper (Tfh) cells and regulatory T (Treg) cells, as well as between naïve CD8+ T cells and exhausted CD8+ T cells (**Supplementary Figure 6A**).

Further analysis of cell composition similarity revealed a high degree of similarity in cell annotation types among the same histological subtypes, except for the LEP+ACI histological pattern in patient P5 (**Figure 3B**). Significant variations in cell proportions were observed across different histological subtypes. In the ACI subtype, CAFs had the highest proportion, while endothelial cells were the most abundant in normal tissue (N). In the MIP subtype, the proportion of CAFs was lower, whereas the proportion of macrophages was higher. Specifically, myofibroblastic CAFs (mCAFs) were the predominant CAF subtype in ACI regions, while M2-like macrophages (e.g., expressing CD163 and MRC1) showed increased prevalence in MIP regions. Statistical analysis confirmed a significant decrease in total CAF fractions (Wilcoxon rank-sum test, p < 0.01) and a significant increase in total macrophage fractions (Wilcoxon rank-sum test, p < 0.005) when comparing MIP regions to ACI regions across all samples. Interestingly, in the MIP+ACI histological pattern, the proportion of CAFs was lower than that in ACI but higher than in MIP, placing it between the two (**Figure 3C**). **Figure 3D** provides a clearer comparison of the proportions of CAFs across the four histological patterns mentioned above. Analysis of CAF marker expression revealed significantly higher co-expression of MMP11, ACTA2 (SMA), and COL1A1 in spots annotated as CAFs within MIP and MIP+ACI regions compared to ACI or normal regions (p < 0.01, Wilcoxon rank-sum test; representative images showing spatial expression of these markers in **Supplementary Figure 8**). These results reveal the heterogeneity of LUAD histological subtypes and the differing enrichment of cells within these subtypes.

We performed enrichment analysis on differentially expressed genes in regions containing micropapillary and acinar histological patterns. The analysis revealed that in the micropapillary regions of all five specimens, pathways related to Extracellular Matrix Organization and Signaling by Receptor Tyrosine Kinases were significantly upregulated (**Supplementary Table S5**). Spatial projection of the GSVA scores for the ‘Extracellular Matrix Organization’ pathway (Reactome: R-HSA-1474244) and ‘Signaling by Receptor Tyrosine Kinases’ pathway (Reactome: R-HSA-9006934) confirmed their heightened activity within the pathologist-annotated MIP regions across the samples (representative visualizations in **Supplementary Figure 9A**). Violin plots of GSVA scores for these pathways by histological region also demonstrated significantly higher enrichment in MIP compared to ACI or N regions (p < 0.01, Wilcoxon rank-sum test, **Supplementary Figure 9B**). GO functional enrichment analysis identified 20 pathways that were upregulated in the micropapillary histological pattern. KEGG functional enrichment analysis revealed four upregulated pathways. REACTOME analysis showed that the Extracellular Matrix Organization pathway was significantly upregulated in micropapillary tissues. In both GO and KEGG analyses, the Focal Adhesion pathway was also significantly upregulated. REACTOME analysis revealed significant enrichment of RTK signaling pathways. The upregulation of the Proteoglycans in Cancer pathway in KEGG analysis also suggests the role of proteoglycans in the metastasis of micropapillary LUAD cells.

**Figure 4:**
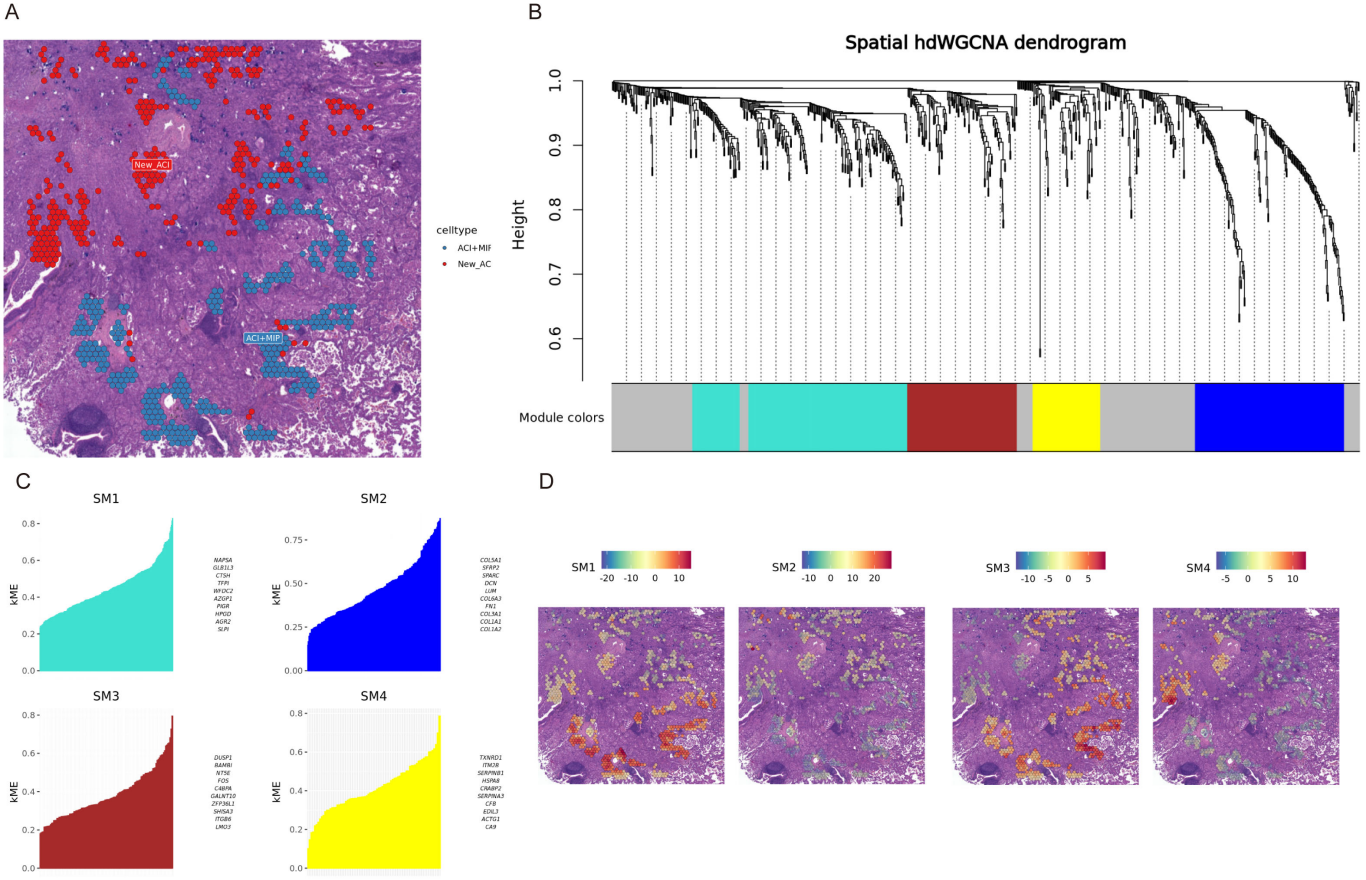
hdWGCNA reveals gene modules associated with histological patterns in patient P1. **(A)** Spatial map of patient P1 H&E stained tissue showing regions predominantly composed of Acinar (ACI, blue spots) and new ACI (New_ACI, red spots identified by module expression, likely corresponding to MIP based on text). **(B)** Dendrogram of hdWGCNA for patient P1, showing gene clustering and identified modules (SM1-SM4) colored below. **(C)** Bar plots showing kME (module membership) scores for the top genes in modules SM1, SM2, SM3, and SM4 for patient P1. **(D)** Spatial visualization of module eigengene scores for SM1, SM2, SM3, and SM4 overlaid on the P1 tissue section. Color intensity indicates module expression level.

Considering that the heterogeneity of LUAD tissue also exists within the defined regions, where different histological patterns may intersect, we individually analyzed each spot from the samples. This allowed us to precisely identify spots containing only MIP pattern cells and those containing only ACI pattern cells for further analysis (**Figure 4A**). The lepidic (LEP) pattern present in patient P5 was not included in this specific WGCNA comparison because the primary focus was on the transition and differences between the more aggressive MIP pattern and the intermediate ACI pattern, which were more consistently represented across multiple samples. Additionally, the limited number of spots exclusively containing LEP in P5 might not provide sufficient statistical power for robust module detection in comparison to MIP and ACI. Given that these spots were fewer in number, the differential analysis had considerable uncertainty. We employed Weighted Gene Co-expression Network Analysis (WGCNA) to identify gene modules and calculated the odds ratios for the overlap of each module with cell type marker genes. A higher odds ratio indicates a stronger significance of overlap between the genes within the module and the marker genes of a specific cell type. Patient P1 was clustered into four gene modules: SM1, SM2, SM3, and SM4 (**Figure 4B**, P1 dendrogram), showcasing the most representative genes for each module (**Figure 4C**). For patient P1, the SM1 and SM3 modules significantly overlapped with genes from micropapillary growth regions, while the genes in the SM2 and SM4 modules significantly overlapped with genes from ACI regions (**Figures 4A, D**). We summarized that module P1.SM1, P1.SM3, P2.SM1, P2.SM2, P2.SM4, P3.SM1, P3.SM3, P4.SM1, P5.SM2, and P5.SM3 significantly overlapped with representative genes from the corresponding micropapillary regions (**Figure 4A**, **Supplementary Figure 6B**). Based on the differences in gene modules, we conducted enrichment analysis, revealing that the BH3 Domain Binding (GO:0051434) pathway was enriched in the relevant modules of four specimens (P1.SM1, P2.SM1, P3.SM3, P5.SM3). The Extracellular Matrix Organization (GO:0030198) and Focal Adhesion pathways were enriched in the relevant modules of three specimens.

### Heterogeneity of dedifferentiation states in different histological patterns

**Figure 5:**
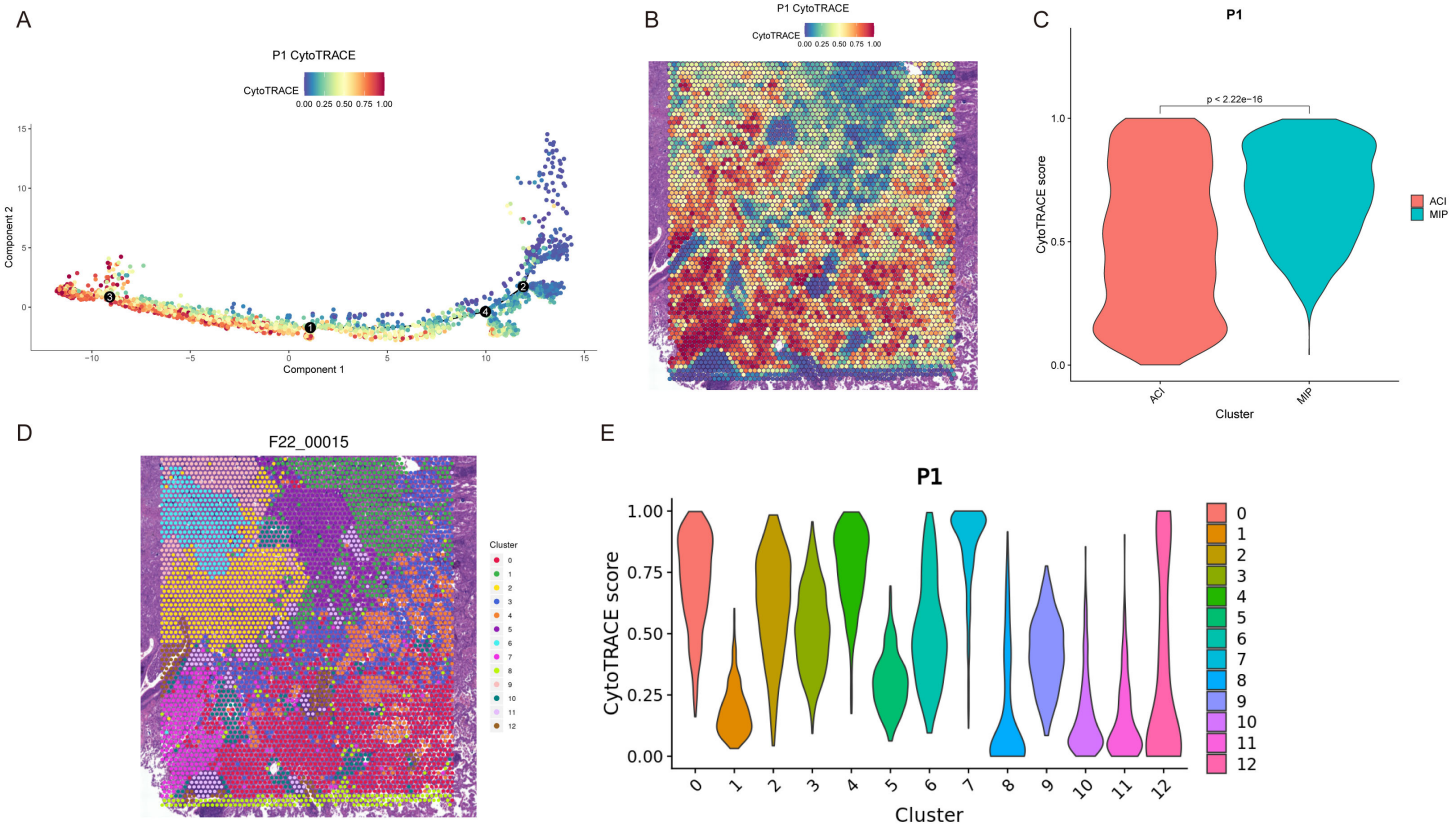
Dedifferentiation trajectory analysis reveals heterogeneity in LUAD subtypes. **(A)** Pseudotime trajectory of spots from patient P1, colored by CytoTRACE score. Black circles indicate branch points. **(B)** Spatial map of patient P1 showing CytoTRACE scores overlaid on tissue spots. Higher scores (blue/purple) indicate greater dedifferentiation potential. **(C)** Violin plot comparing CytoTRACE scores between ACI and MIP regions in patient P1 (p < 2.22e-16). **(D)** Spatial map of patient P1 (F22_00015, likely an internal ID for P1) colored by Seurat clusters (0-12). **(E)** Violin plots showing CytoTRACE scores for each Seurat cluster (0-12) in patient P1.

To gain deeper insight into the dynamic dedifferentiation changes of specific subtypes, we performed differentiation analysis using Monocle (27) and CytoTRACE (26) and applied principal component analysis to visualize developmental trajectories (**Figure 5A**). Patient P1 included two histological subtypes, MIP and ACI, and we observed that the MIP region exhibited higher CytoTRACE scores compared to the ACI region (**Figures 5B, C**). Within the cluster of the ACI region, cluster 2 (**Figure 5D**) was located at the boundary of the ACI area, with a CytoTRACE score higher than other clusters within ACI but lower than those in the MIP region (**Figure 5E**). Patient P3 included MIP+ACI, ACI, and normal (N) regions, with the MIP+ACI region showing higher CytoTRACE scores compared to the ACI region, while both were higher than the normal tissue region (**Supplementary Figure 10**). Across all five patients (P1-P5), a quantitative comparison revealed that regions with a higher proportion of MIP pattern consistently exhibited significantly higher mean CytoTRACE scores compared to regions predominantly composed of ACI or LEP patterns (ANOVA, p < 0.001). Furthermore, there was a significant positive correlation between the percentage of MIP pattern within a tumor region (as determined by pathological annotation) and its average CytoTRACE score (Pearson r = 0.78, p < 0.01 across all samples). For patient P1 (**Figures 5D, E**), detailed analysis of the 12 identified clusters showed distinct gene expression signatures. For instance, clusters predominantly in the MIP region (e.g., clusters 8, 9, 10 with high CytoTRACE scores) were enriched for genes involved in cell proliferation (e.g., MKI67, TOP2A) and EMT (e.g., VIM, ZEB1). Conversely, clusters mainly in the ACI region (e.g., clusters 0, 1, 3 with lower CytoTRACE scores) showed higher expression of differentiation markers (e.g., NKX2-1, SFTA1P) and pathways related to cell adhesion. Cluster 2, with an intermediate CytoTRACE score, co-expressed markers from both MIP-associated and ACI-associated clusters, suggesting a transitional state. A full list of differentially expressed genes and pathway enrichments for each cluster is provided in **Supplementary Tables S6, S7**.

### Cell communication

To characterize cell-cell communication networks, we used CellPhoneDB (29) to infer potential ligand-receptor interactions. This analysis revealed a complex web of communication, with specific interactions enriched in different histological contexts (detailed interactions in **Supplementary Figure 11**). Tumor cells exhibited strong interactions with CAFs, primarily involving TGFB1/2 (Tumor) - TGFBR1/2 (CAF) and various collagen-integrin pairs, particularly enriched in MIP and ACI regions. CAFs, in turn, interacted broadly with immune cells through CXCL12 (CAF) - CXCR4 (various immune cells) signaling. Notably, we found a high number of predicted interactions between M2-like macrophages and cDC2_CD1C cells, including pairs like CCL5-CCR5 and CD86-CTLA4, which were predominantly found within MIP regions across multiple samples.

**Figure 6:**
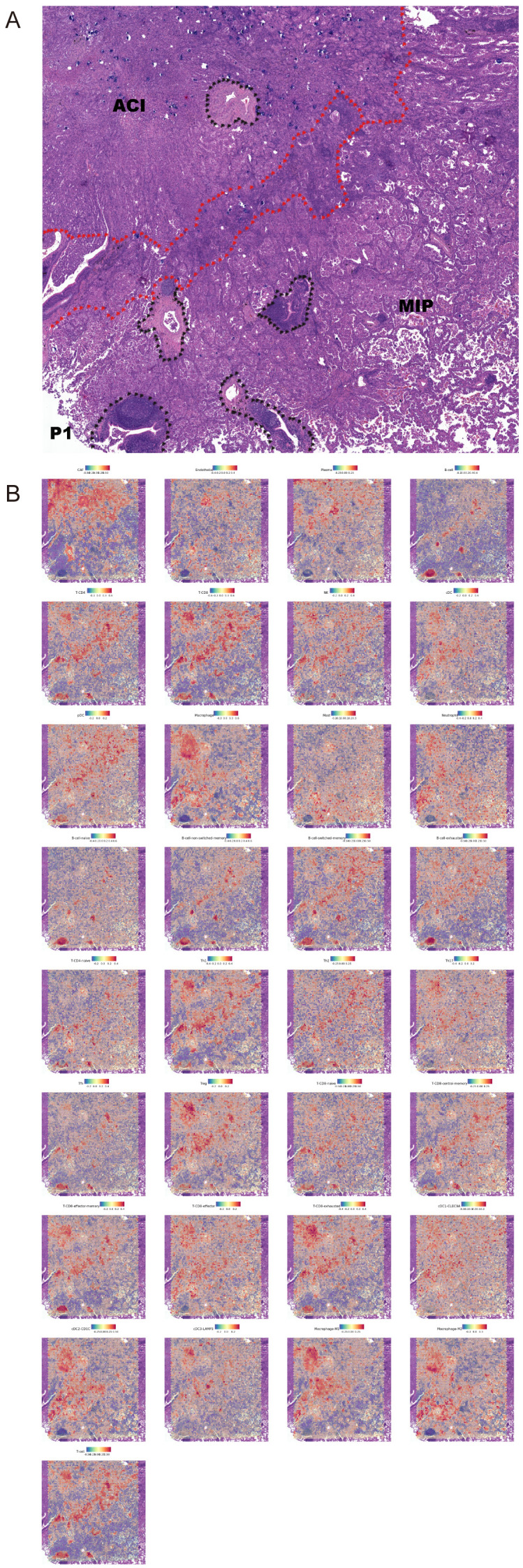
Spatial distribution of annotated cell types in a representative LUAD sample. **(A)** Hematoxylin and eosin (H&E) stained tissue section from patient P1 showing distinct acinar (ACI) and micropapillary (MIP) histological regions. The boundary is indicated by the red dotted line. Black dotted outlines highlight tertiary lymphoid-like structures. **(B)** Spatial feature plots showing the distribution and relative abundance scores of 33 annotated cell types across the tissue section from patient P1. The color scale for each plot indicates low (blue) to high (red) abundance. Note the marked enrichment of Macrophages, exhausted T-CD8 cells, and cDC2_CD1C cells within the MIP region.

To visualize the spatial context of these cellular ecosystems, we mapped the distribution of all annotated cell types in a representative patient (P1), which presents clear ACI and MIP histological regions (**Figure 6A**). The resulting spatial feature plots reveal distinct cellular compositions that align with the histological patterns (**Figure 6B**). For instance, consistent with our quantitative analysis, Macrophage abundance is visibly higher in the MIP region compared to the ACI region. Similarly, cell types such as exhausted T-CD8 cells and cDC2-CD1C cells show a clear enrichment within the MIP area. This spatial co-localization of M2-like macrophages and cDC2_CD1C cells within the MIP microenvironment provides a structural basis for the extensive interactions predicted by CellPhoneDB. Comparison of interaction strengths revealed that MIP regions generally showed higher overall interaction scores for pathways related to immune modulation and ECM remodeling compared to ACI or normal regions. We observed that when M2 macrophages served as ligand cells, CD74_COPA, CD74_APP, and CD74_MIF were present in the analysis results of every specimen.

## Discussion

The molecular characteristics of tumors and their microenvironment determine tumor occurrence and progression (30). Recent studies have indicated that driver mutations in LUAD are not associated with specific histological subtypes, and oncogenic alterations do not drive subtype progression or spatial heterogeneity. Epigenetic and transcriptional reprogramming are key determinants of histological subtypes (7). Meanwhile, substantial progress has been made in RNA-seq, proteomics, and single-cell profiling; however, information regarding the spatial localization of tumor cells, stromal cells, and immune cells is lost during tissue dissociation. The lack of comprehensive spatial features in tumors remains a barrier to improving therapeutic strategies and clinical prognosis (31). Therefore, delineating histological characteristics at spatially resolved molecular resolution is crucial for describing the heterogeneity of tumor histological subtypes and their corresponding molecular features. Here, we integrated spatial transcriptomics and histomorphology in invasive LUAD to elucidate the molecular mechanisms driving histological subtype progression and microenvironment composition.

Our findings indicate that histological subtypes exhibit high similarity in major cell types, but the degree of cell enrichment varies among different subtypes. Studies have shown that CAF-mediated paracrine TGFβ signaling induces tumor tissue remodeling and determines the histological patterns of LUAD, leading to tumor heterogeneity (32). Additionally, characterizations of CAFs have revealed that different phenotypes of CAFs are significantly associated with the prognosis of lung cancer patients (33). M2 macrophages can modulate T helper cells (Th), B cells, and cytotoxic T cells (Tc) in their surrounding tumor microenvironment, thereby influencing patient prognosis (34). The upregulation of the Extracellular Matrix Organization pathway suggests a crucial role of ECM in the progression of micropapillary lung adenocarcinoma. ECM remodeling is often closely associated with tumor cell invasion and metastasis, and the high invasiveness of the micropapillary histological pattern may partially be attributed to the active state of this pathway (35). The upregulation of RTK signaling indicates that tumor cells in this region may have acquired enhanced proliferation and invasive capabilities through RTK-mediated signaling pathways. The activation of RTKs has been linked to poor prognosis and drug resistance in various cancers, further supporting the aggressive characteristics of micropapillary LUAD (36). These results suggest that dynamic changes in cell adhesion and ECM remodeling in the tumor microenvironment may be important mechanisms driving the invasion and metastasis of lung adenocarcinoma. Tumor cells gain enhanced migratory and invasive abilities by modulating their adhesion to the ECM, which is particularly pronounced in micropapillary lung adenocarcinoma. Receptor Tyrosine Kinases (RTKs) signaling is often regulated through Focal Adhesion Kinase (FAK). FAK is a non-receptor tyrosine kinase located in focal adhesions, involved in mediating downstream effects of RTK signaling. FAK promotes tumor cell proliferation, survival, and migration by activating signaling pathways such as PI3K/AKT and MAPK (37, 38). The upregulation of the Proteoglycans in Cancer pathway in KEGG analysis also suggests the role of proteoglycans in the metastasis of micropapillary LUAD cells, further emphasizing the regulatory role of proteoglycans in RTK signaling (39). The process of cellular dedifferentiation has been implicated in tumor progression (40). The histological subtypes of invasive LUAD are associated with prognosis, with high-grade histological subtypes, including micropapillary (MIP), showing significantly increased risks of recurrence and metastasis (8, 20). These subtypes are also related to clinical responses to immune checkpoint inhibitor treatments (41). Regions with higher CytoTRACE scores suggest that the tumor cells in those areas possess stronger undifferentiated characteristics or tumor stemness (26). These cells retain higher self-renewal capabilities, which may be related to tumor invasiveness and drug resistance, and often include cancer stem cells, the presence of which is typically associated with risks of tumor recurrence and metastasis (42, 43).

The type, structure, and function of cells, as well as their ability to change morphology and function under different conditions, are fundamental factors determining tissue morphology formation and development. Most lung adenocarcinomas are characterized by the coexistence of two or more histological subtypes within the tumor tissue. Therefore, a comprehensive study of cellular lineage composition is crucial. We found that histological subtypes exhibit high similarity in major cell types, but the degree of cell enrichment varies among different subtypes. However, studies have suggested that cell composition is inferred from a human lung comprehensive cell atlas containing 58 cell subpopulations, which reveals extensive plasticity of cell types and cell type-specific gene expression during organ evolution, providing important molecular data and insights into the behavior of lung cells in different biological contexts (44). Other novel or rare cell types, especially intermediate cell states, may not be resolvable or identifiable with current technologies. To overcome this limitation, we used an unsupervised framework to predict the differentiation status of each spatial point and observed heterogeneity in dedifferentiation states between histological subtypes, consistent with histological morphology. Interestingly, a recent study using genetically engineered mouse models of human cancer revealed highly plastic cell states (HPCS) during the progression of lung cancer. HPCS cells exhibit robust differentiation and proliferation potential (45). Importantly, the MP subtype demonstrated significantly heterogeneous differentiation states, which may explain the emergence of micropapillary histological morphology.

In our study, CAFs were the most prevalent cell type. This may indicate their important roles in immune responses, phagocytosis, angiogenesis, and lung homeostasis. The heterogeneity of CAFs is increasingly recognized as a critical factor in the tumor microenvironment. For instance, Pellinen et al. utilized multiplex fluorescence immunohistochemistry to identify distinct CAF subsets in non-small cell lung cancer (NSCLC), demonstrating that specific subsets (CAF7 and CAF13, defined by markers like PDGFRA, PDGFRB, FAP, and αSMA) had opposing associations with tumor histology, driver mutations, immune features, and patient prognosis (46). Cords L et al. showed that CAFs can be divided into 11 phenotypes: ifnCAFs (IDO+), tCAFs (CD10+/CD73+), hypoxic tCAFs (CAIX+/CD10+), iCAFs (CD34+/CD248+), vCAF (CD146+/CD34-), dCAFs (Ki-67+), SMA CAFs (SMA+/FAP?/MMP?/Collagen)?, hypoxic CAFs (CAIX+), and PDPN CAFs (PDPN+). Collagen-expressing CAFs are categorized into mCAFs (MMP11+/SMA+/Collagen+) and collagen CAFs (Collagen+/FN+/MMP11-/SMA)?. Their findings indicated that mCAFs and collagen CAFs are enriched in solid and micropapillary histological patterns (33). In their study, mCAFs and collagen CAFs were associated with poorer prognosis. Our research also validated this, showing that the gene signature corresponding to MMP11+/SMA+/Collagen+ mCAFs expression in the MIP region and MIP+ACI region was higher than in other regions. The development of comprehensive marker panels, such as the 42-marker panel for imaging mass cytometry described by Røgenes et al. for studying CAF niches in breast cancer, underscores the importance of deeply characterizing CAF heterogeneity and their interactions with immune and cancer cells (47). The complex interplay between these diverse CAF subtypes and immune cells is a critical determinant of tumor progression and response to therapy, as specific CAF populations can recruit or exclude immune cells, modulate their function, and contribute to an immunosuppressive microenvironment, thereby impacting immunotherapy efficacy (48). Further investigation into the spatial organization and functional states of distinct CAF populations in LUAD subtypes is warranted.

Secondly, there are many macrophages in the tumor microenvironment (TME), followed by T cells, including naive T cells, effector T cells (T helper cells and cytotoxic CD8+ T lymphocytes), and memory T cells (central memory T cells and effector memory T cells), which mediate adaptive immune responses. Notably, we found different TAM subpopulations among the various histological subtypes, indicating that TAM subpopulations play an indispensable role in reshaping the tumor environment and mediating immunosuppression to promote tumor progression. Macrophages act as antigen-presenting cells (APCs) by activating T cells through MHC/TCR interactions and co-stimulatory signaling, and they activate anti-tumor immunity by recruiting innate immune cells. For instance, cytotoxic CD8+ T lymphocytes (CTLs) execute effector functions and promote cell death through performing granzyme and Fas/FasL pathways (49). Dysfunctional or exhausted T cells in the TME are characterized by the overexpression of inhibitory checkpoint molecules, such as PD-1, TIM-3, LAG3, CTLA4, and TIGIT.

The molecular mechanisms underlying the progression of LUAD remain unclear. Annotating tumor samples solely based on histological characteristics lacks information about the intratumoral heterogeneity or histological subtypes. Gene regulatory networks, including transcription factors and target genes, determine the transcriptional state of cells. We found that regulatory networks associated with differentiation and proliferation, such as ECM remodeling and RTK signaling pathway activation, modulate cellular plasticity and reprogramming, as well as potential cell state transitions. Furthermore, we observed a significant micropapillary subtype during subtype progression, which is associated with clinical responses to immune checkpoint inhibitor treatment. This subtype exhibits notable undifferentiation and stemness during cellular dedifferentiation. Cells of these subtypes retain stronger self-renewal capabilities, which may be related to the aggressiveness and drug resistance of the tumor. Additionally, these cells may contain cancer stem cells, whose presence is often linked to the risk of tumor recurrence and metastasis (42, 43). In other words, cells with high self-renewal capacity in tumors may lead to more severe disease, as they can support tumor growth and spread, as well as resistance to treatment. Further research is needed to elucidate the relationship between the micropapillary subtype and cellular dedifferentiation.

In summary, our findings demonstrate the dynamic biological changes and dedifferentiation states of tumor subtypes during the progression process. Through spatially resolved molecular profiling, we were able to obtain detailed and objective information about cancer cells and their microenvironment (i.e., tumor microenvironment, TME) during tumor progression. This enables us to directly analyze the molecular characteristics and heterogeneity of cellular dedifferentiation states throughout subtype progression, providing new potential insights for treatment choices. In short, this study reveals important biological processes in tumor development and may offer valuable guidance for future therapeutic strategies.

## Data Availability

The original contributions presented in the study are included in the article/**Supplementary Material**. Further inquiries can be directed to the corresponding author.

## Supplementary material

Supplementary Figure 1 Histological examination of samples from 5 patients with LUAD.

Supplementary Figure 2 A representative image showing the number of genes studied in defined spots of LUAD samples.

Supplementary Figure 3 Clustering into 10 classes according to spatial transcriptomes in spots.

Supplementary Figure 4 Differential gene expression in the identified 10 clusters. The heatmap displays the top 8 differentially expressed genes for each cluster, sorted by log fold change.

Supplementary Figure 5
**(A)** Spatial feature plots illustrating the distribution and relative abundance of annotated cell types across a representative tissue section (Patient P5). Red indicates higher abundance and blue indicates lower abundance for each cell type. **(B)** Cells annotated included different immune cells and other cells types in which CAFs were found to be the majority.

Supplementary Figure 6
**(A)** Significant spatial correlations between different cell types within the same spot. The correlation matrix was generated using data aggregated from all five patients (P1-P5). **(B)** Significant overlapping of the representative module genes with the corresponding micropapillary regions in different patients’ samples.

Supplementary Figure 7 Detailed mapping showing possible microscopic infiltration of tumor cells into adjacent normal tissue in patients 3 to 5.

Supplementary Figure 8 Co-expression of CAF markers COL1A1, ACTA2 and MMP11 was significantly higher in MIP than in ACI as demonstrated by spatial expression analysis.

Supplementary Figure 9
**(A)** Spatial projection of the GSVA scores for Extracellular Matrix Organization and Signaling by Receptor Tyrosine Kinase pathway. **(B)** Violin plots comparing the Gene Set Variation Analysis (GSVA) scores for the ‘Extracellular Matrix Organization’ and ‘Signaling by Receptor Tyrosine Kinase’ pathways across Micropapillary (MIP), Acinar (ACI), and Normal (N) regions from all patients. The y-axis represents the GSVA enrichment score.

Supplementary Figure 10 Patient P3 included MIP+ACI, ACI, and normal (N) regions, with the MIP+ACI region showing higher CytoTRACE scores compared to the ACI region, while both were higher than the normal tissue region.

Supplementary Figure 11 Characterization of immune cell interactions that are associated with CAF expression in tumors. This dot plot visualizes significant ligand-receptor interactions between various cell types. The color of each dot indicates the mean expression level of the interacting partners, while the size of the dot corresponds to the statistical significance of the interaction (p-value).
