# Supplementary material for: Spatial transcriptomics uncovers immune-cell plasticity and dedifferentiation signatures in aggressive lung adenocarcinoma subtypes
Source: Front Immunol. 2025 Aug 21;16:1620886. doi: 10.3389/fimmu.2025.1620886 (PMC12408323; doi:10.3389/fimmu.2025.1620886)
Supplement: Supplementary Figure 1 — Histological examination of samples from 5 patients with LUAD. [file DataSheet1.docx]

**Supplementary figure 1**

**
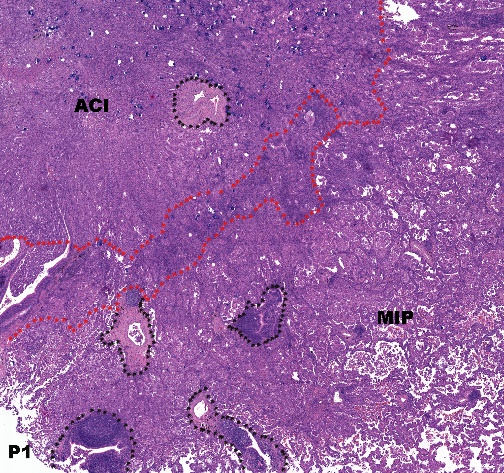

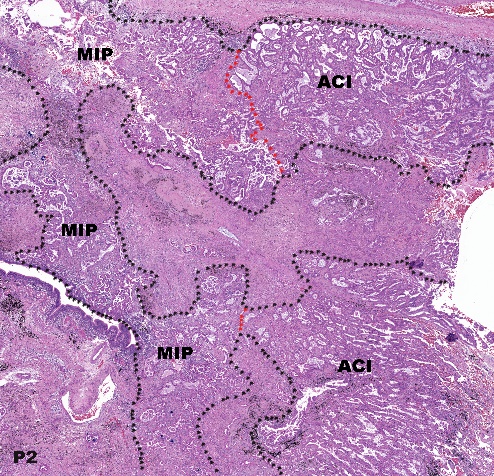
**

**
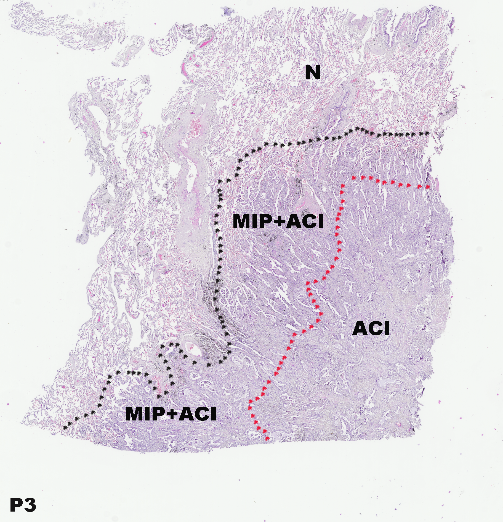

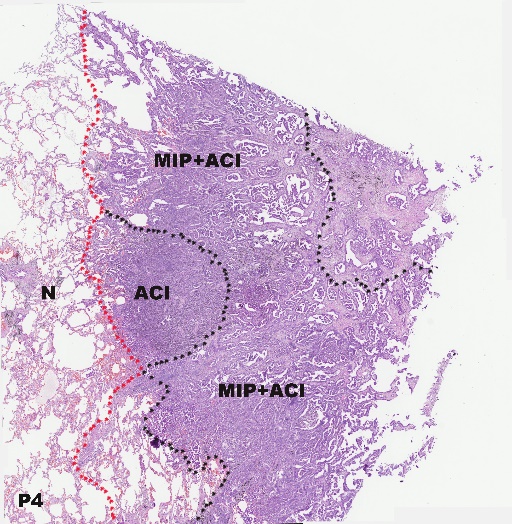
**

**
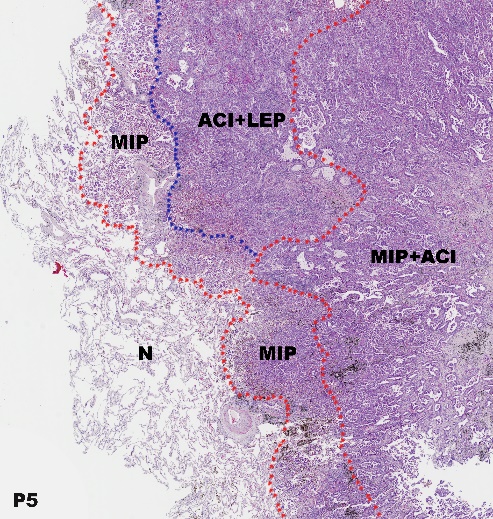
**

**Supplementary figure 2**


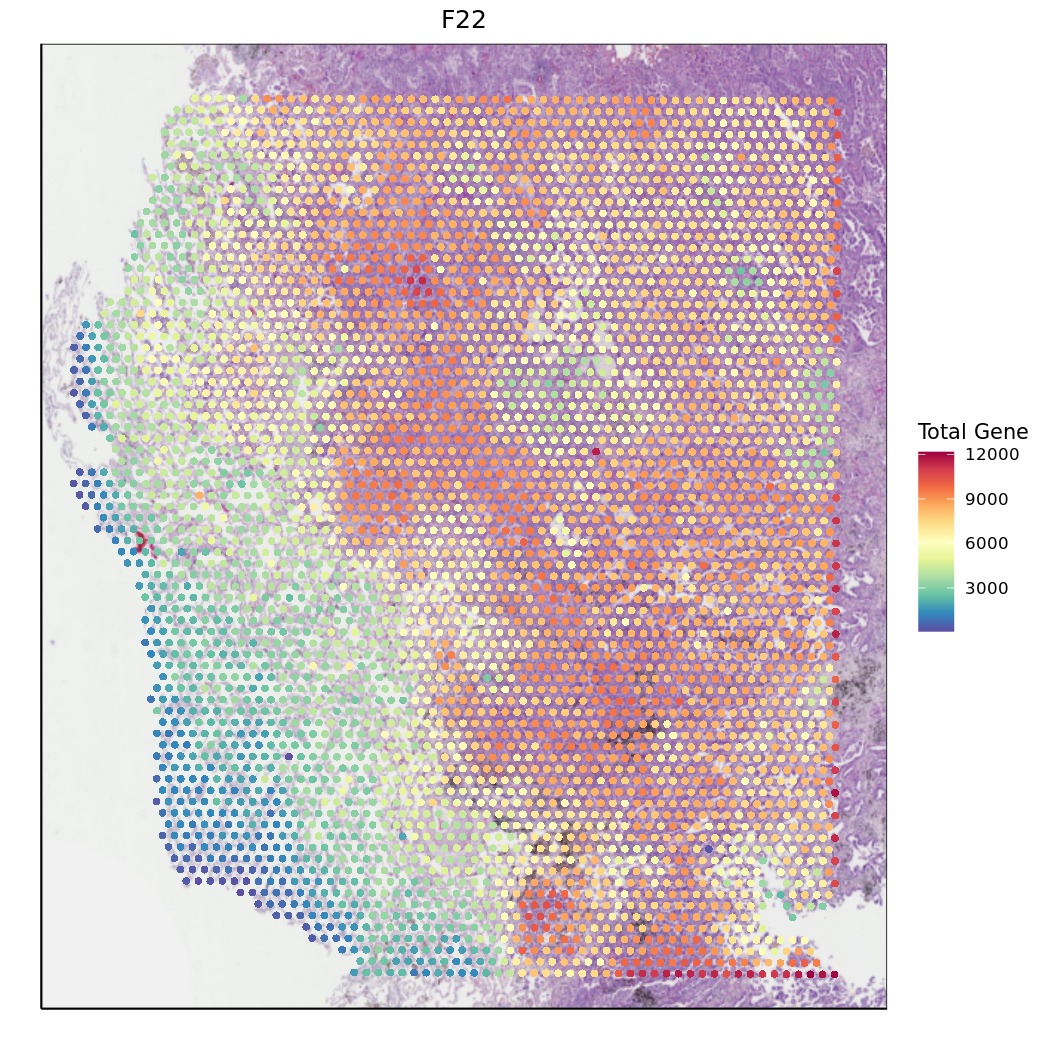


**Supplementary figure 3**

**
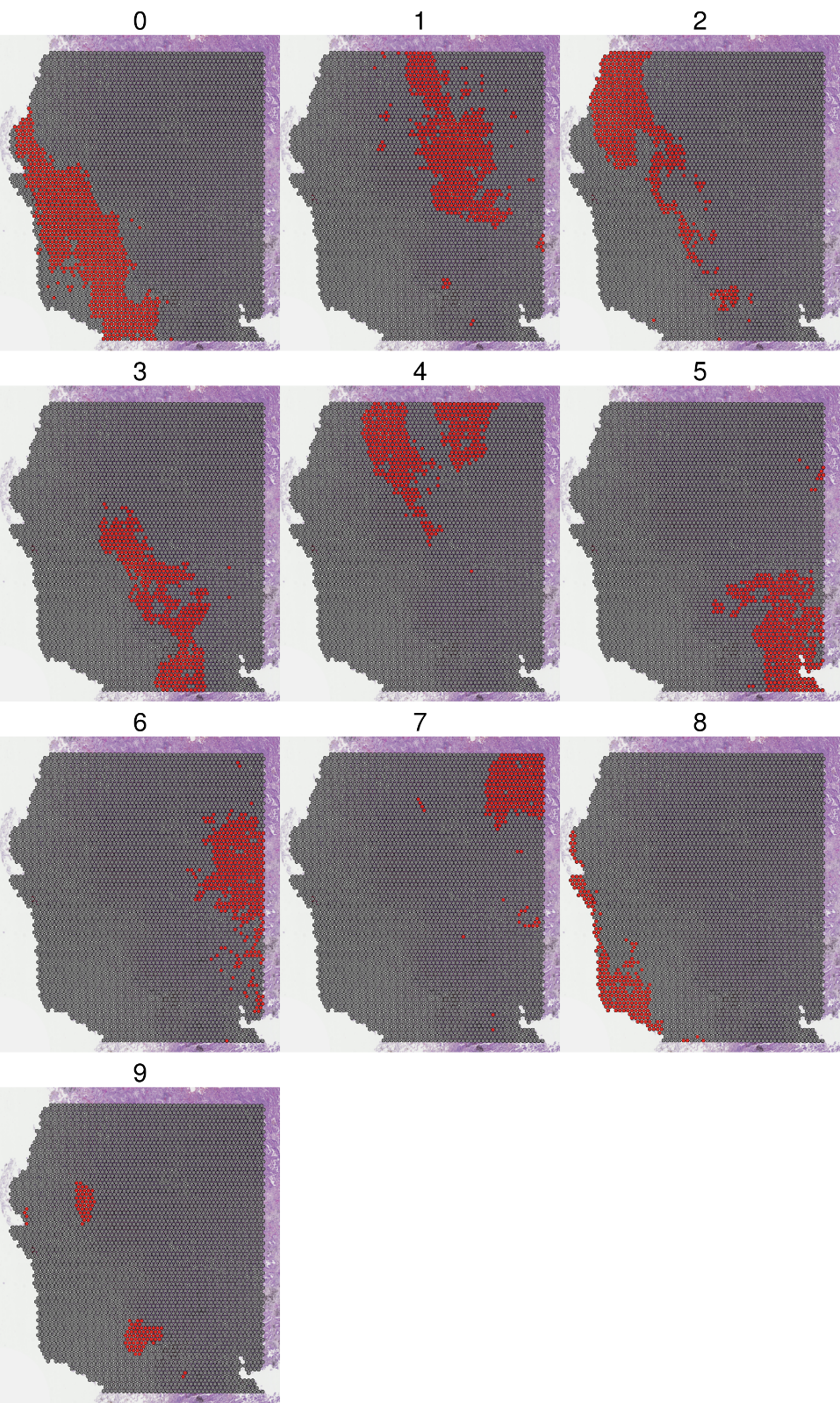
**

**Supplementary figure 4**

Cluster 0 Cluster 1


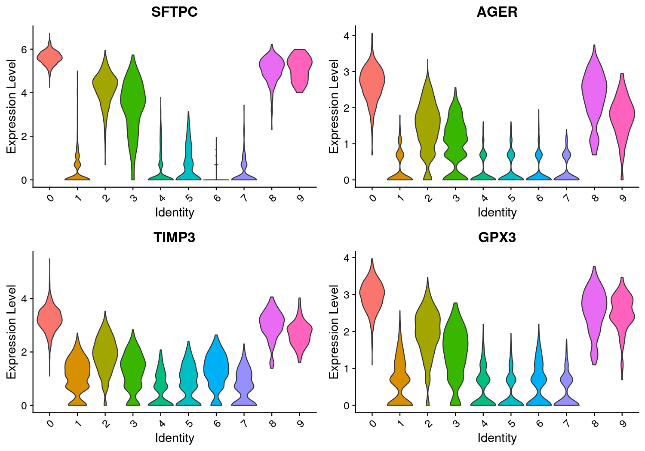

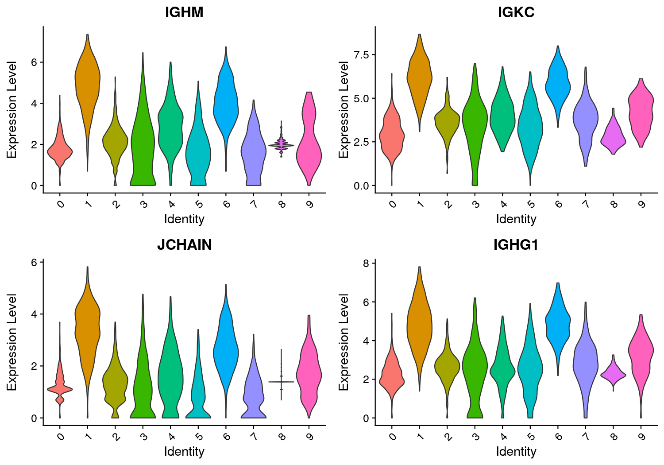


Cluster 2 Cluster 3


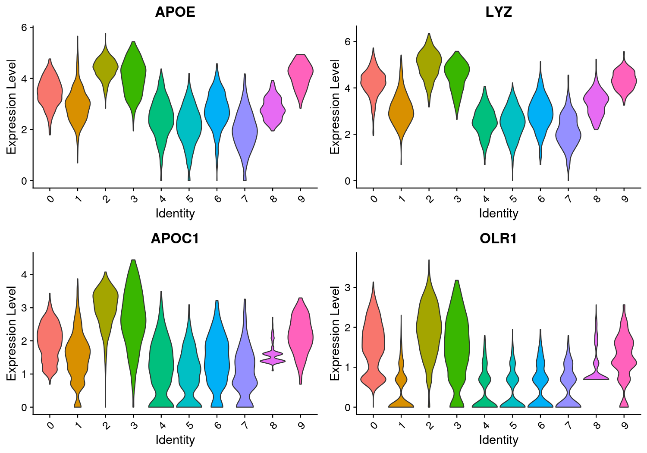

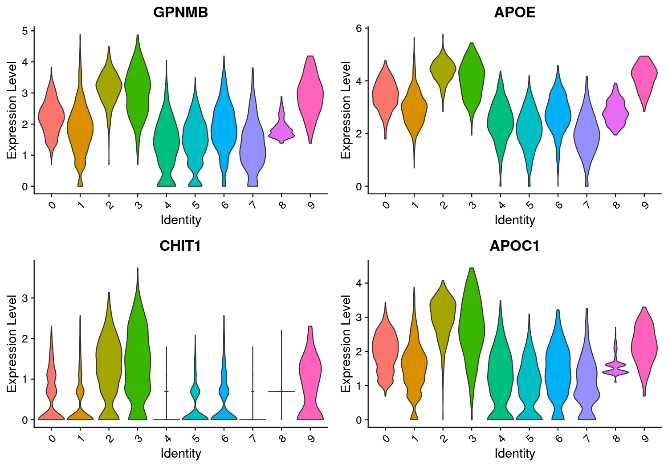


Cluster 4 Cluster 5


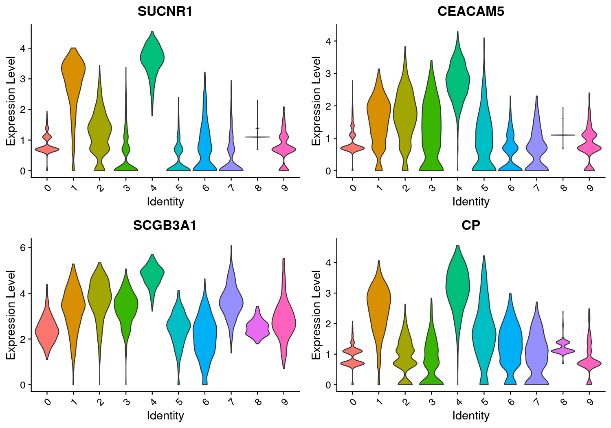

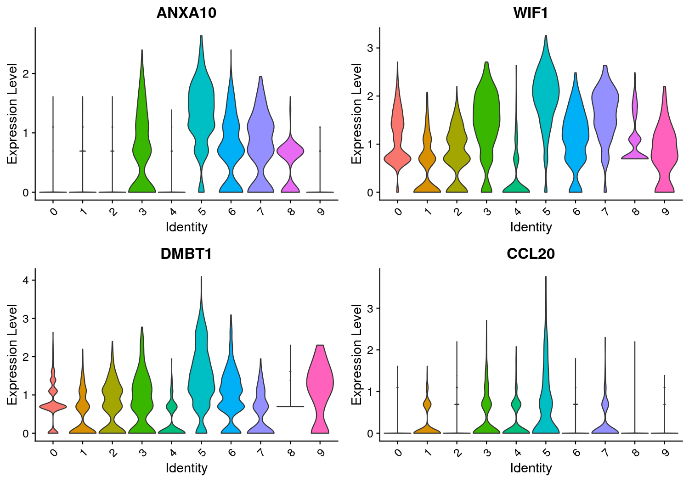


**Supplementary figure 4**

Cluster 6 Cluster 7


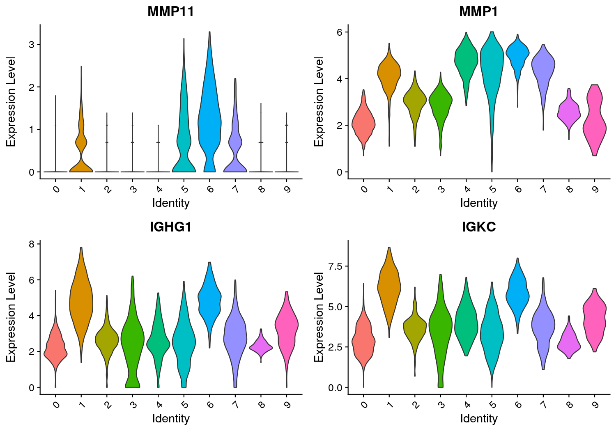

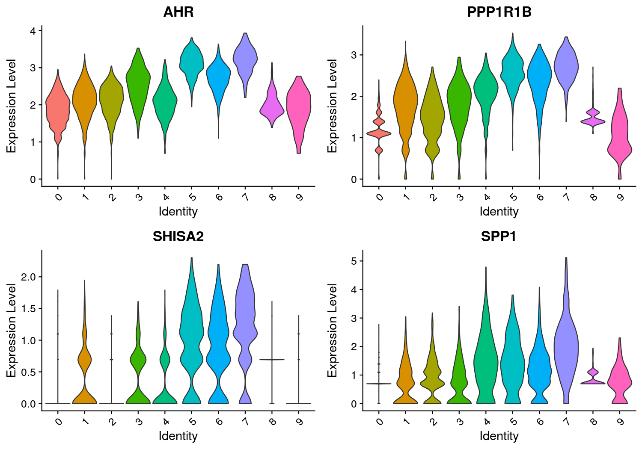


Cluster 8 Cluster 9


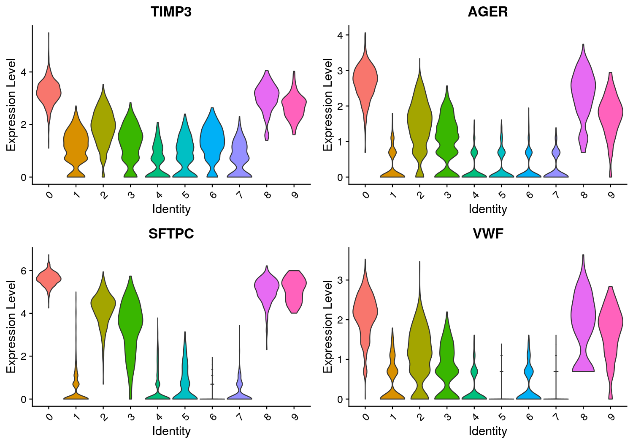

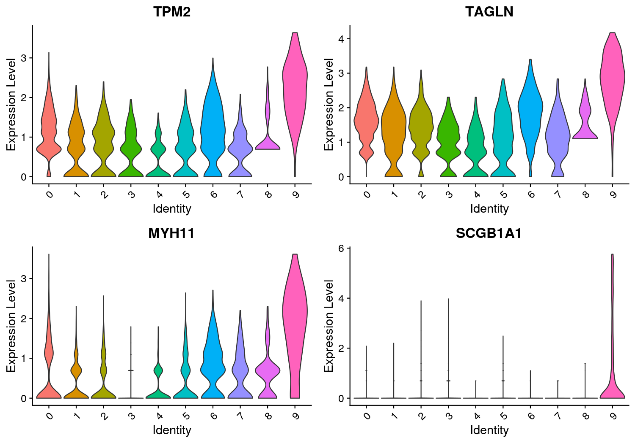


**Supplementary figure 5**

**A**

**
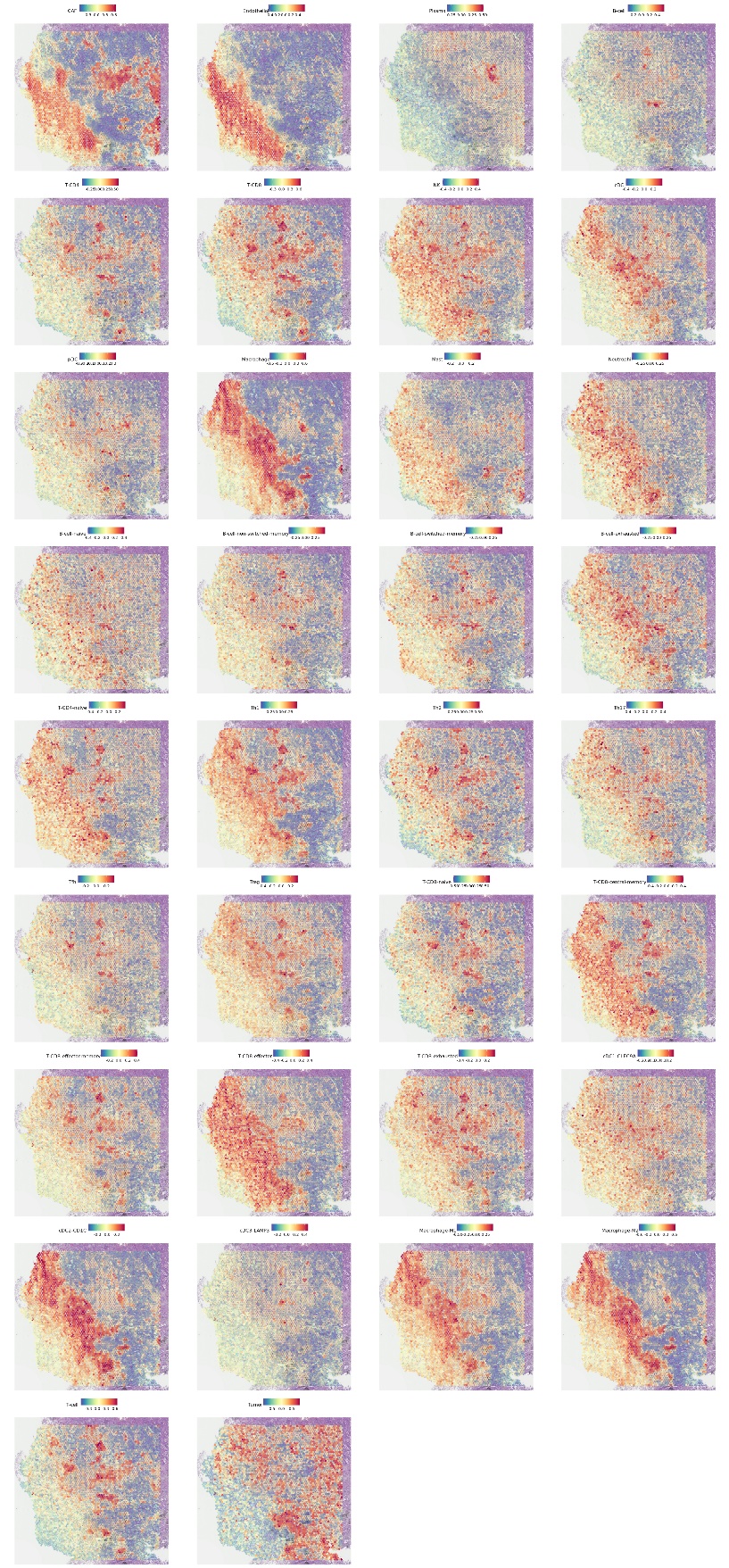
**

**Supplementary figure 5**

**B**

**
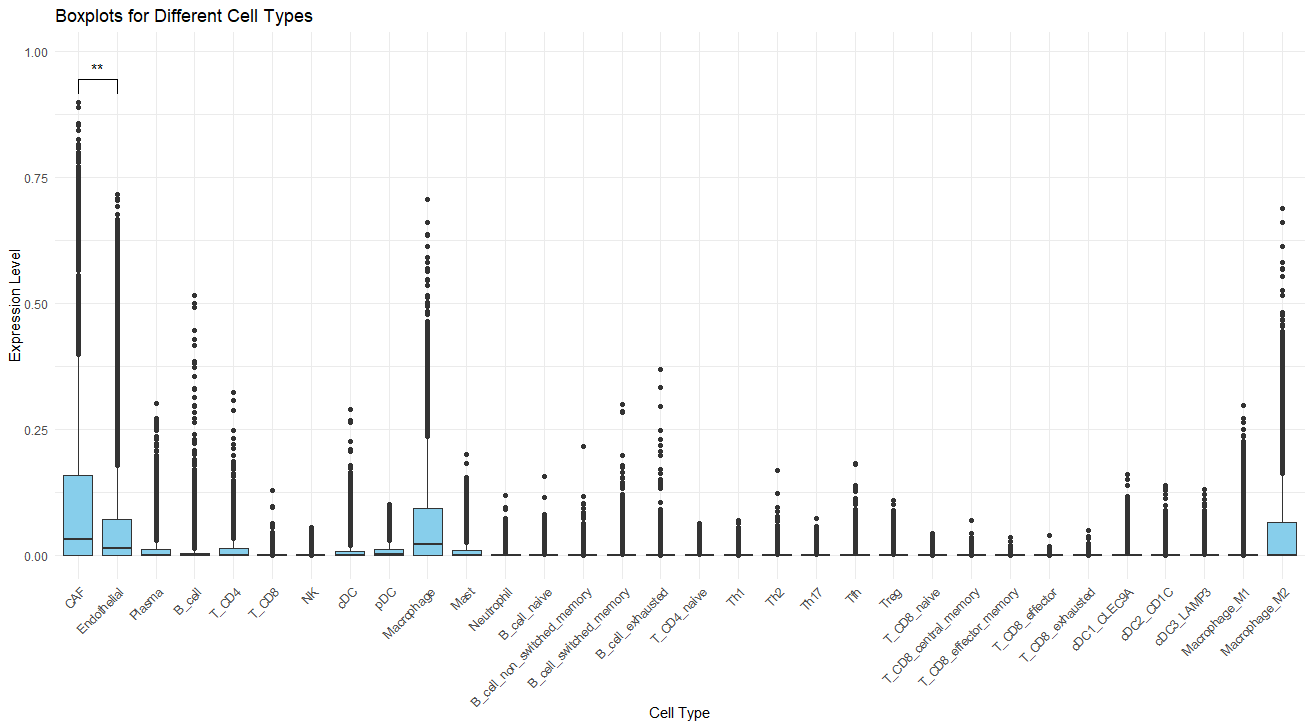
**

**Supplementary figure 6**

**A**

**
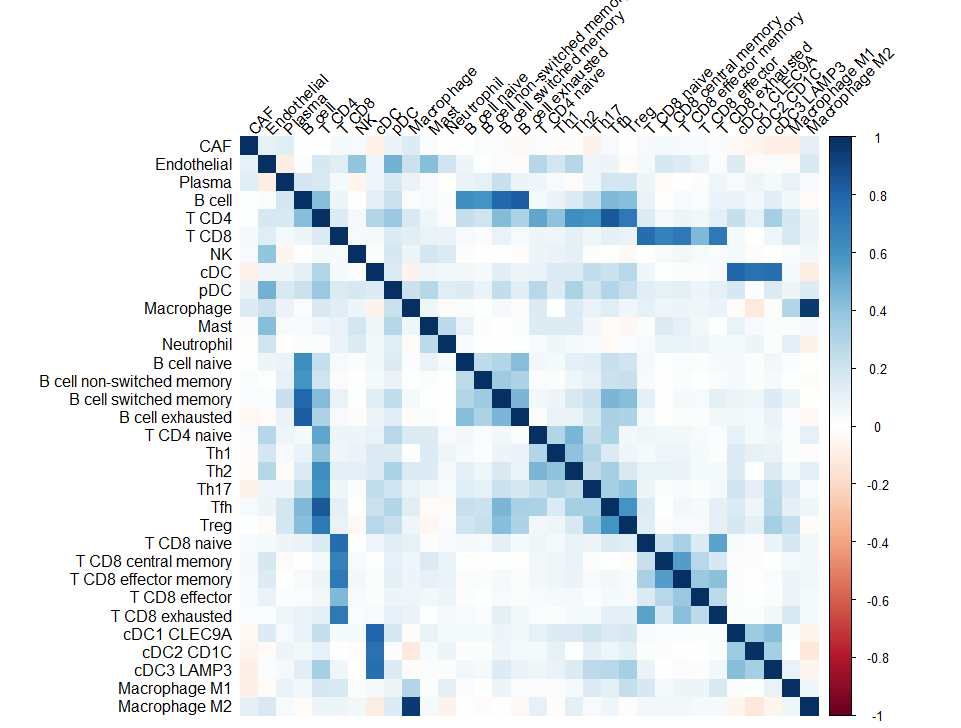
**

**Supplementary figure 6**

**B**

Patient 1

**
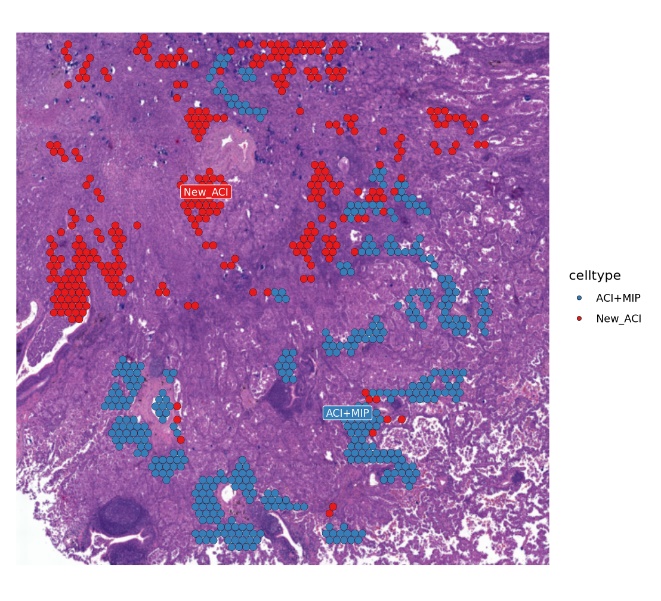

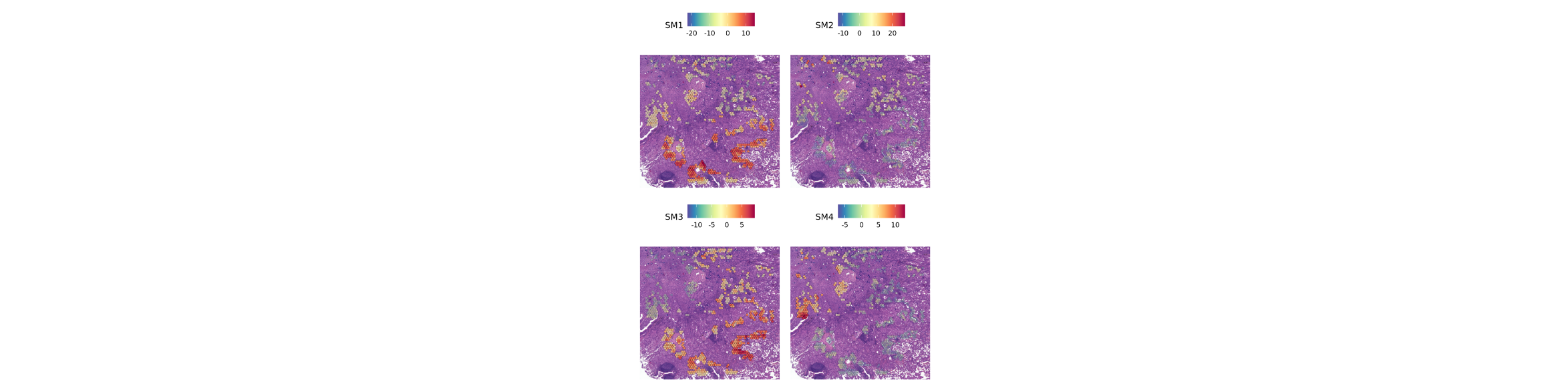
**

Patient 2

**
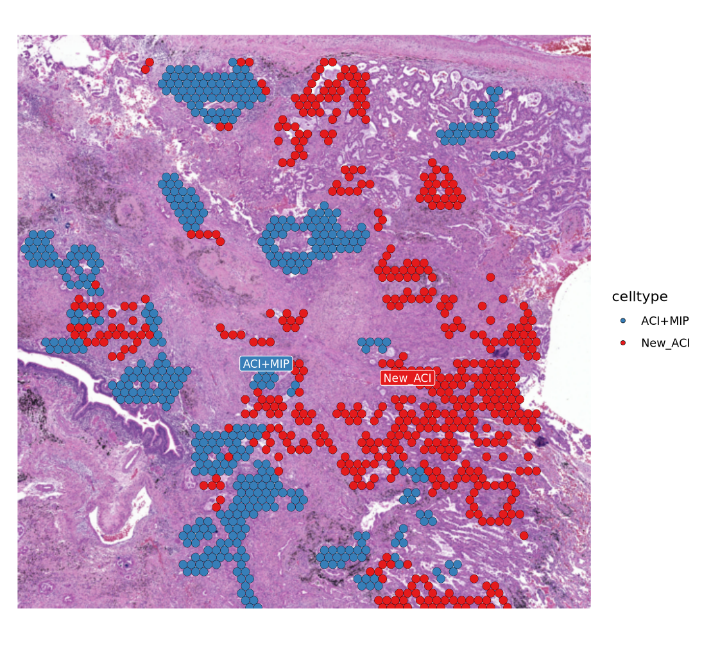

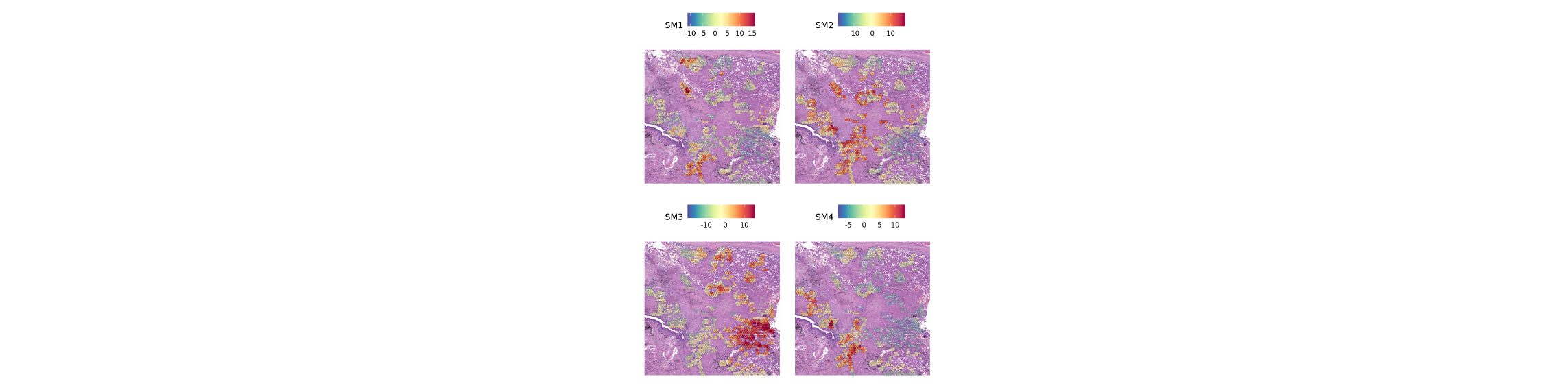
**

**Supplementary figure 6**

**B**

Patient 3


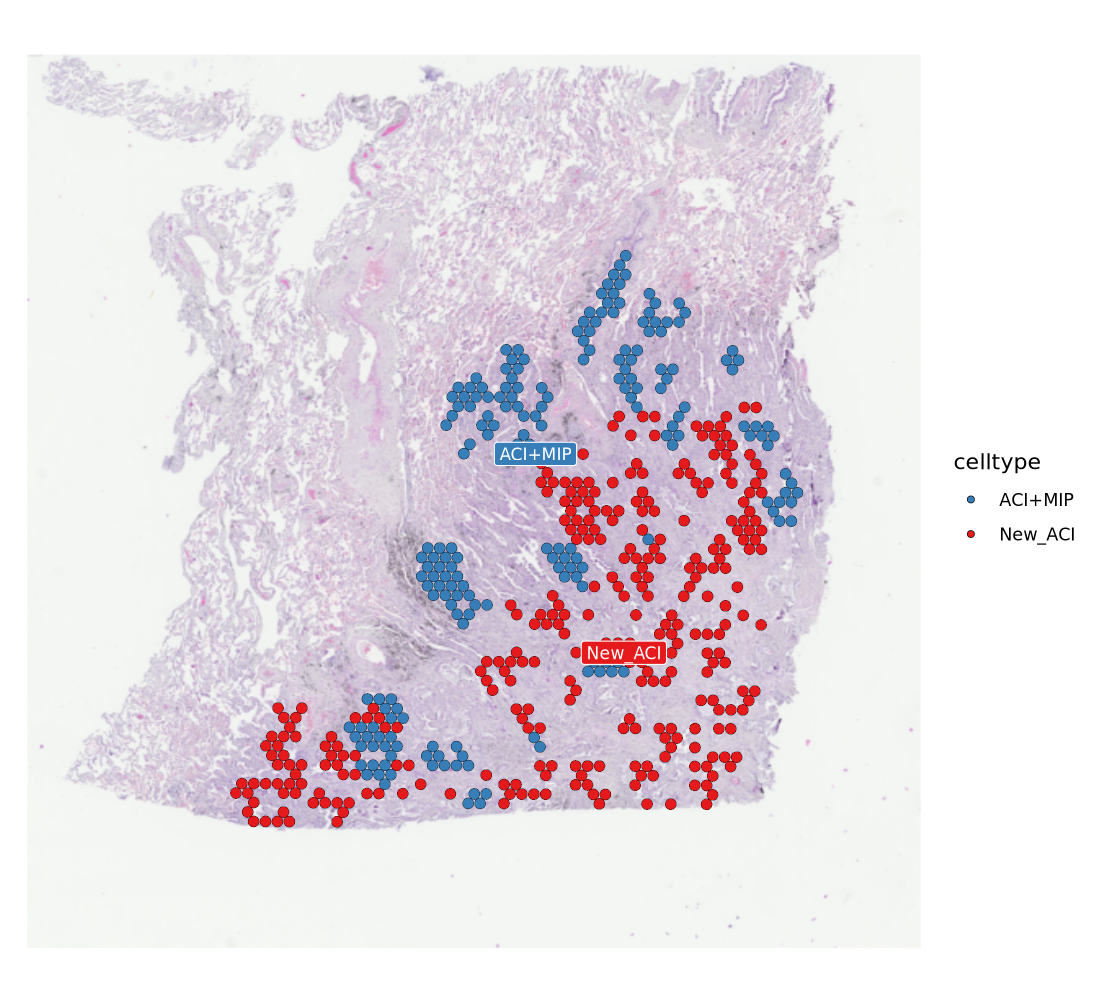

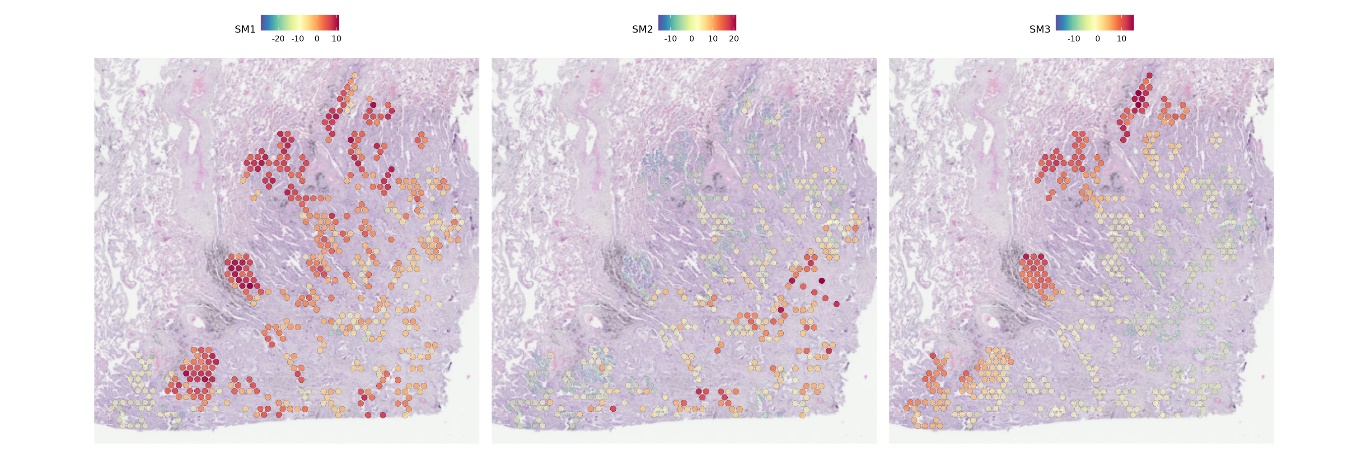


**Supplementary figure 6**

**B**

Patient 4


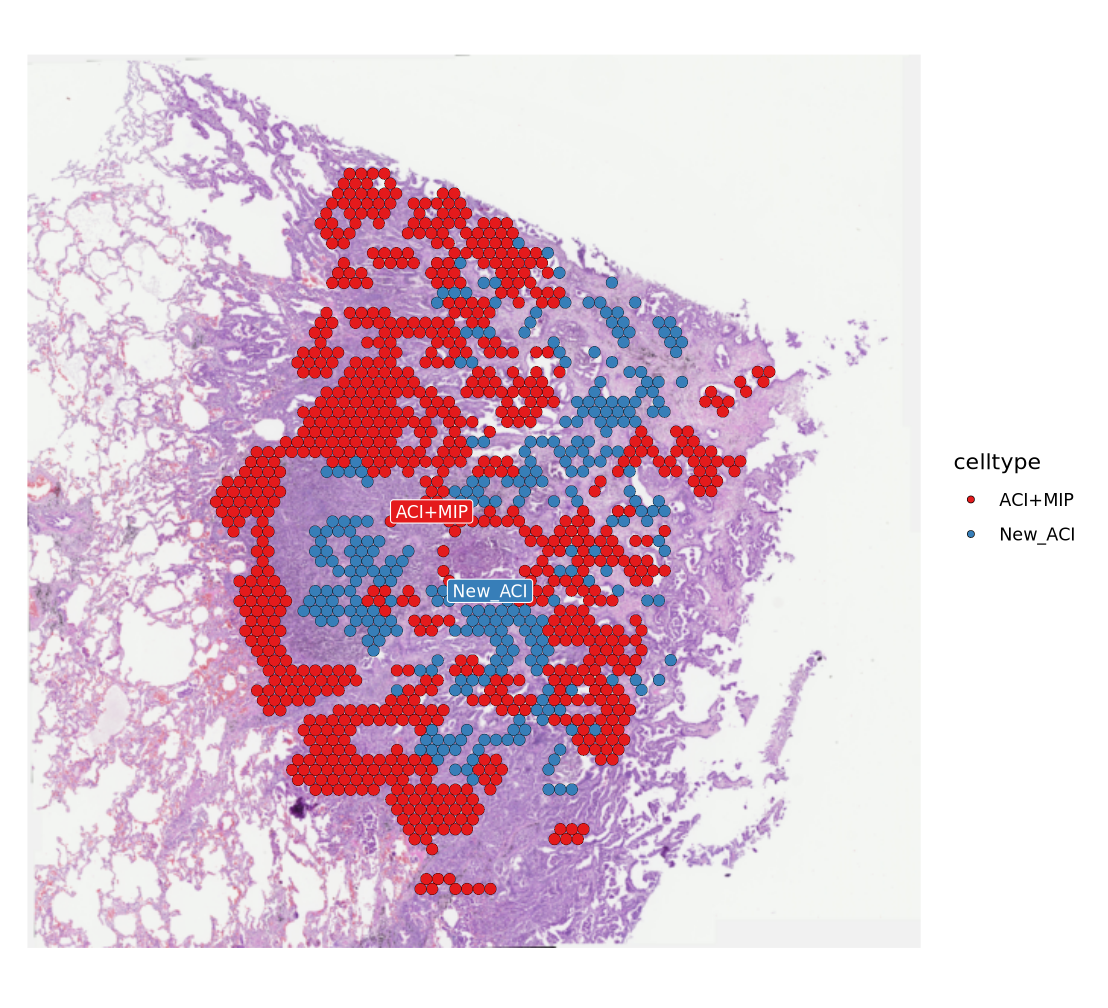


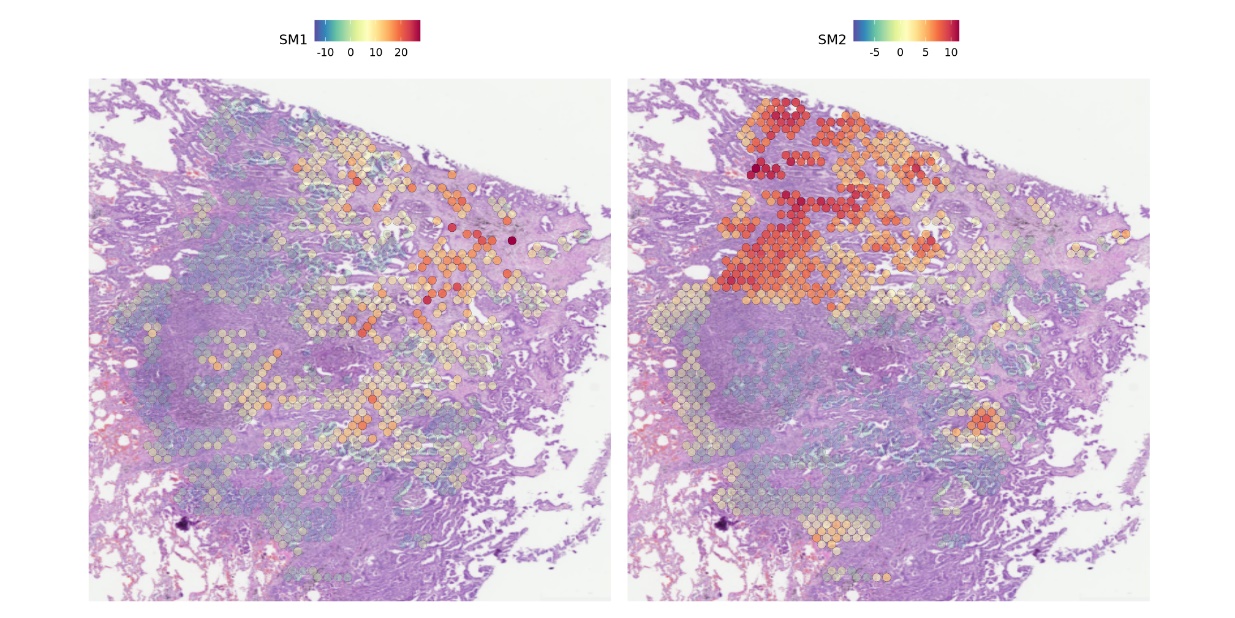


**Supplementary figure 6**

**B**

Patient 5


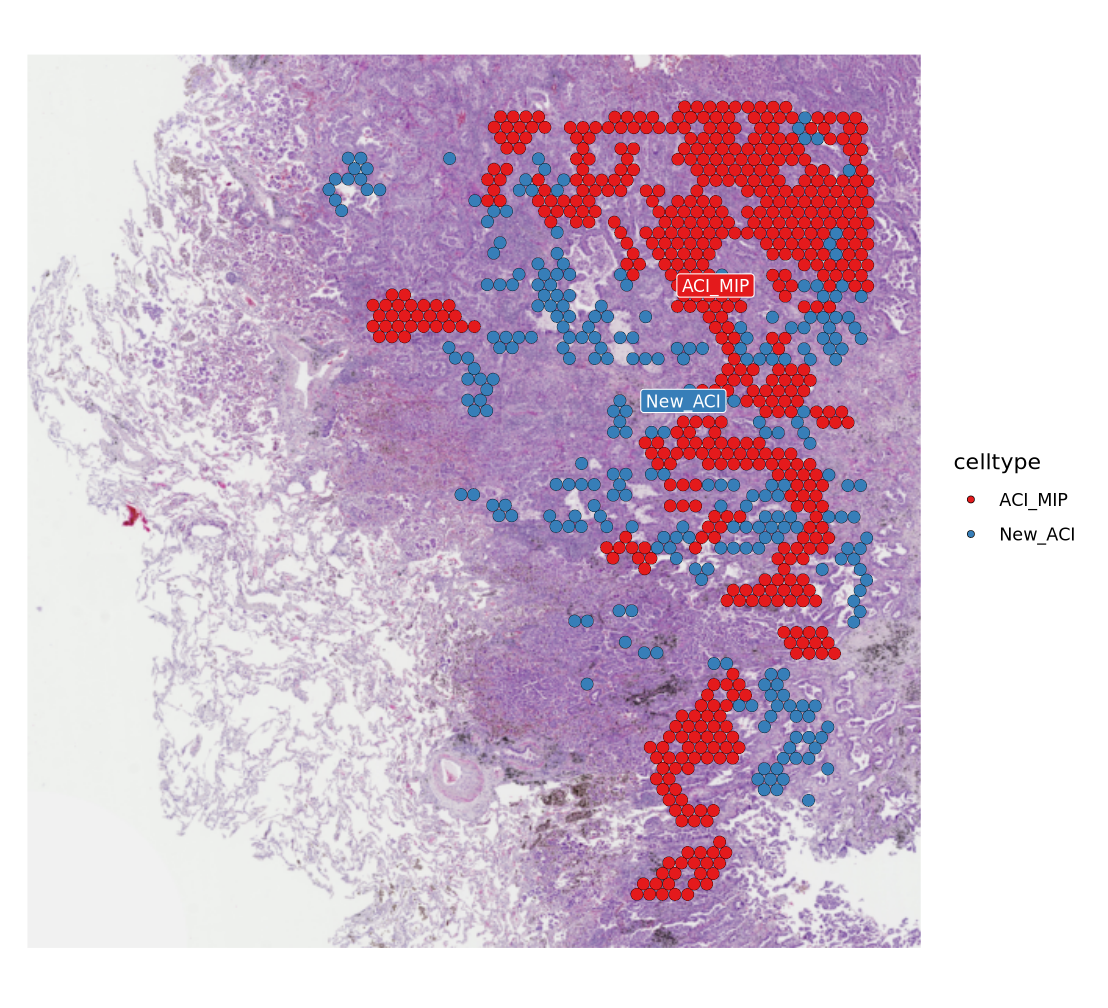

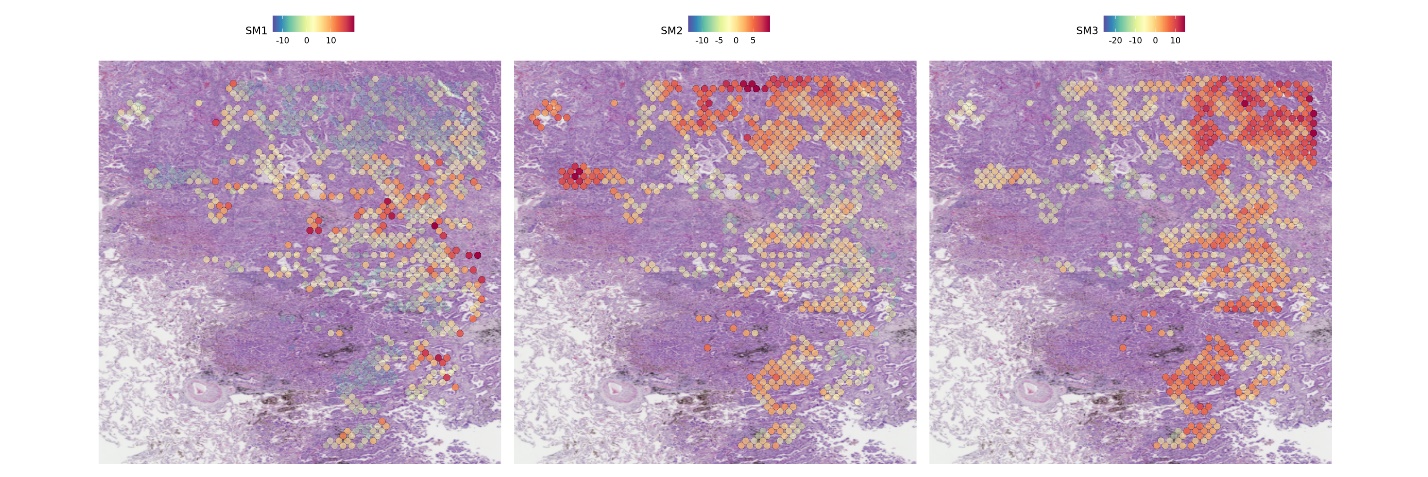


**Supplementary figure 7**

**
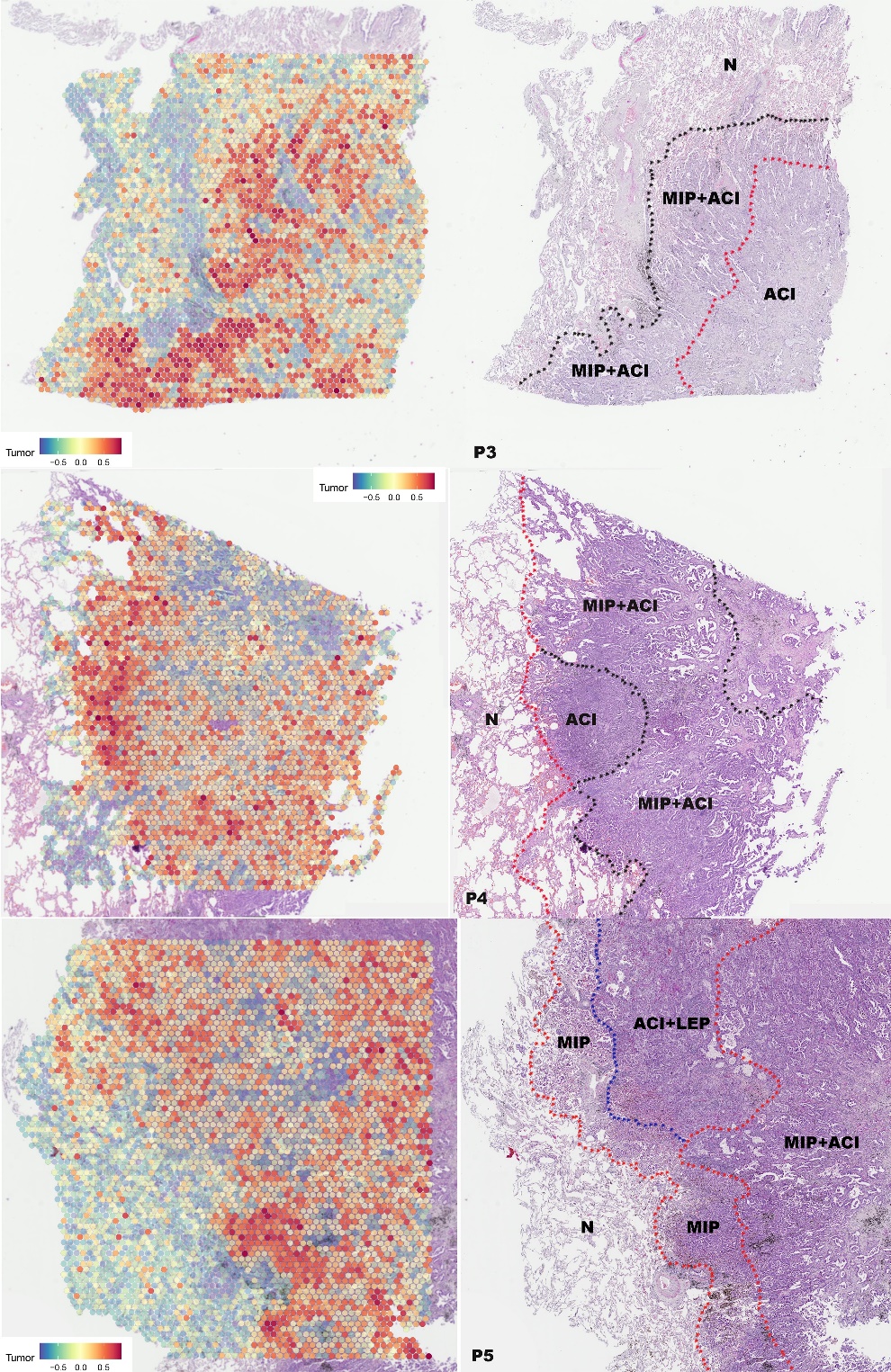
**

**Supplementary figure 8**


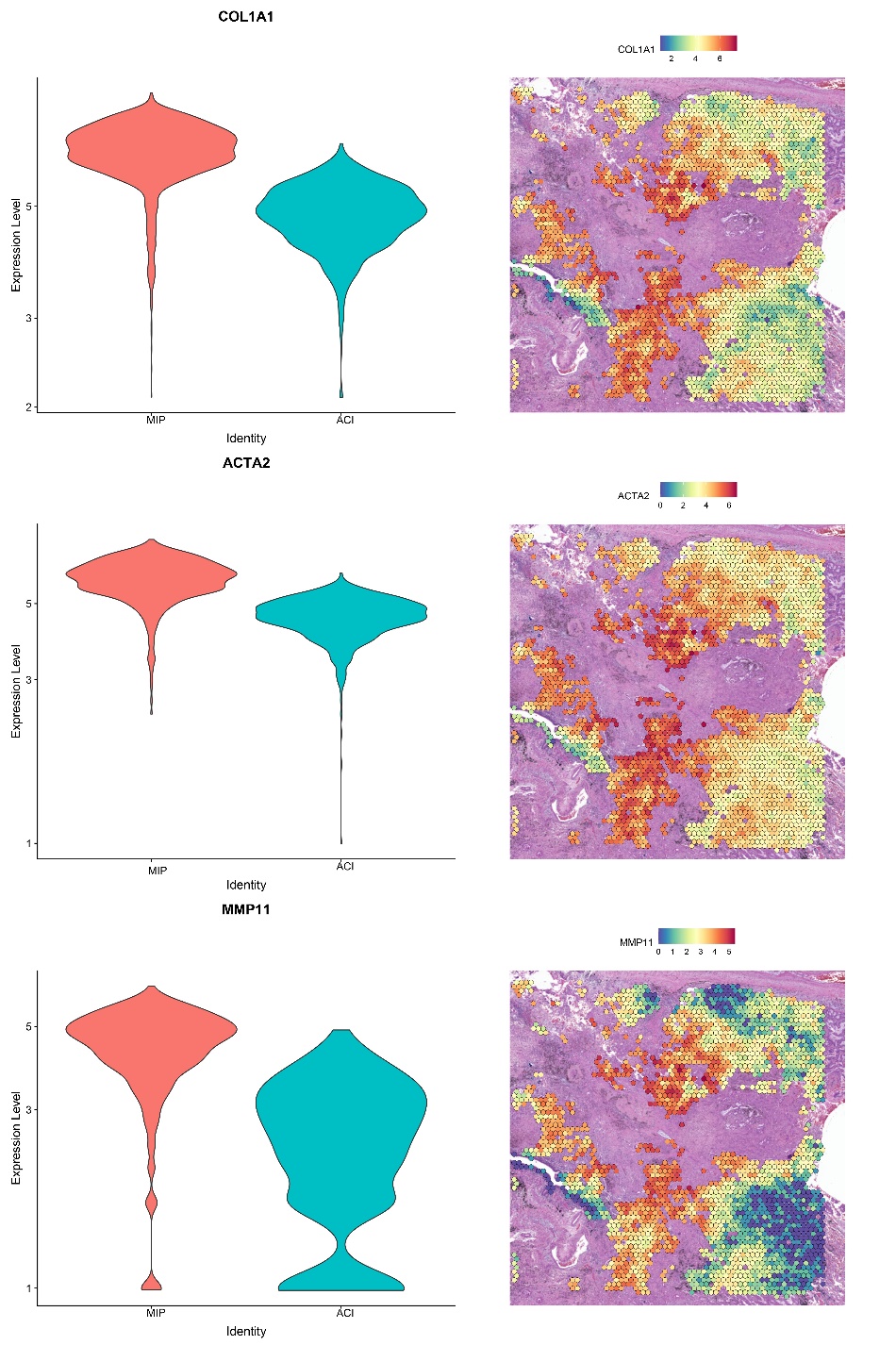


**Supplementary figure 9**

**A**


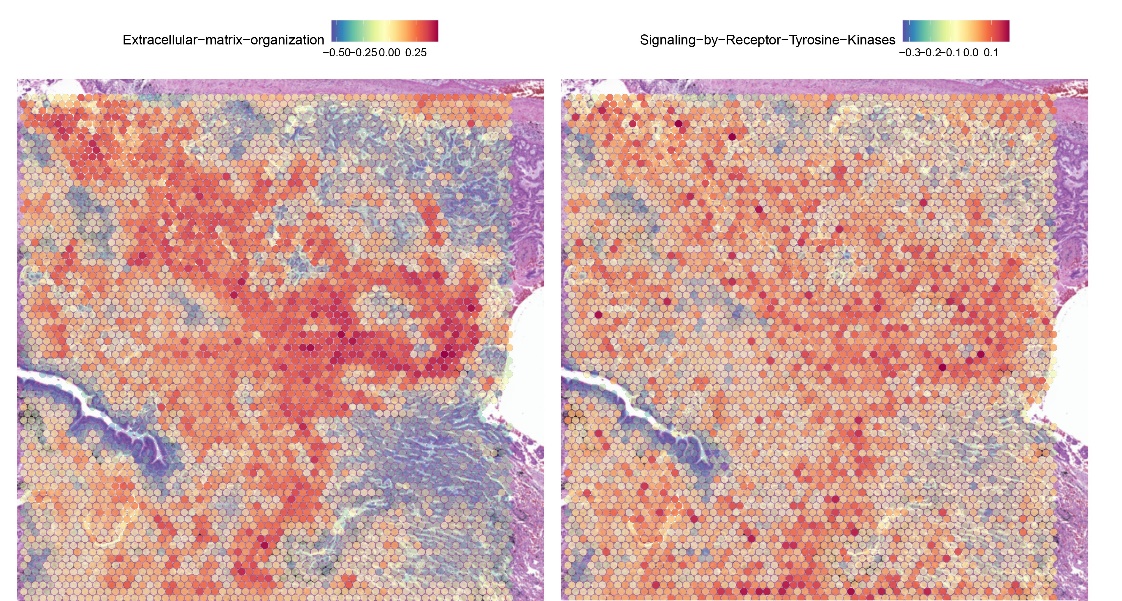


**B**

**
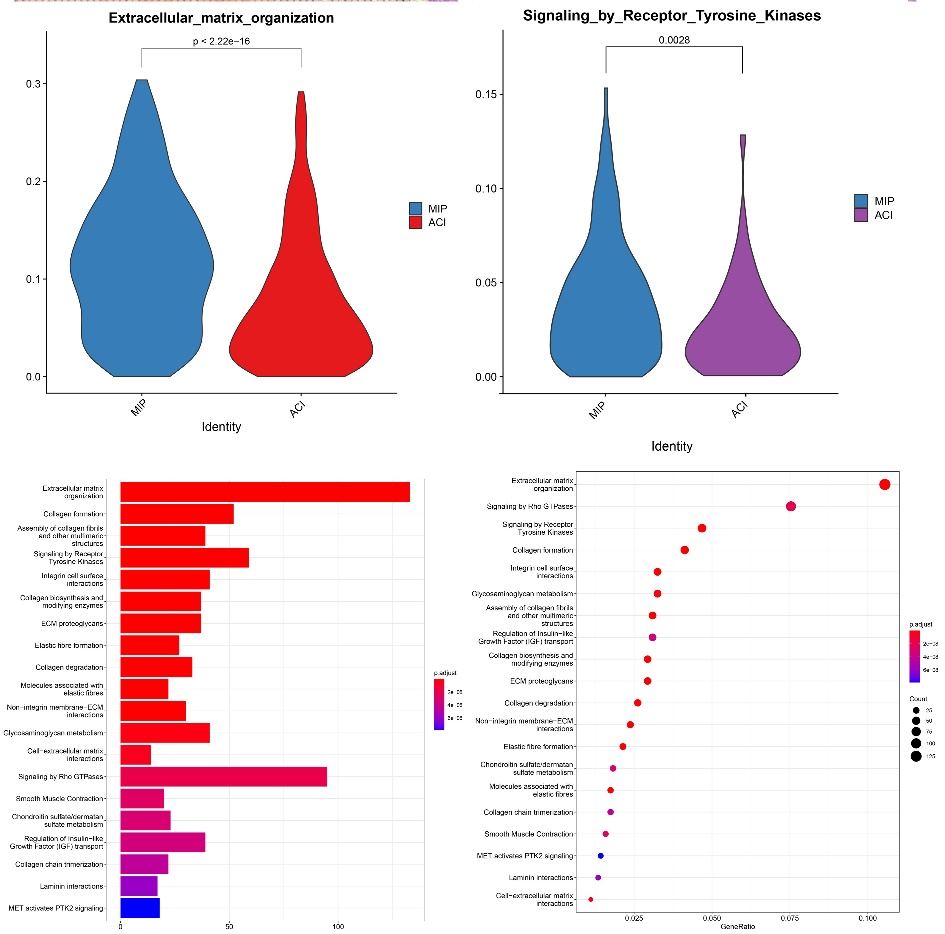
**

**Supplementary figure 10**


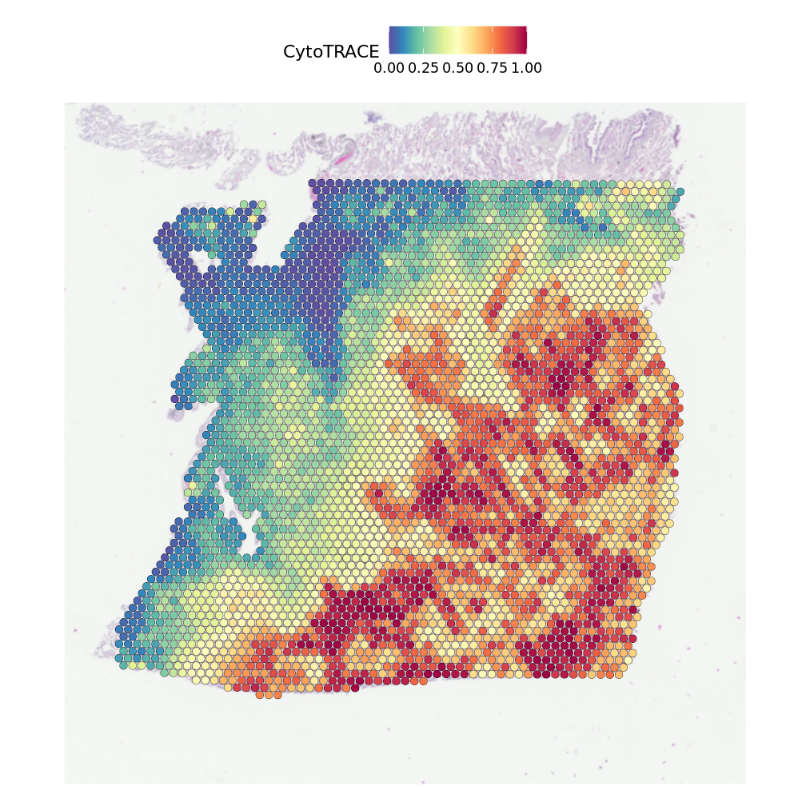


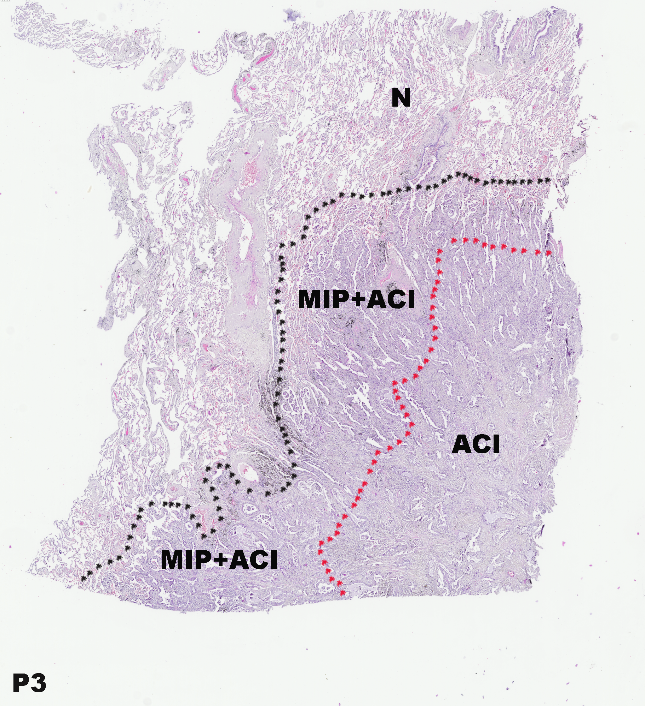


**Supplementary figure 11**

**P1 P2 P3**

**
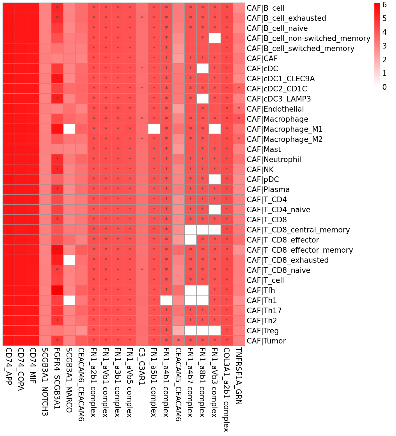

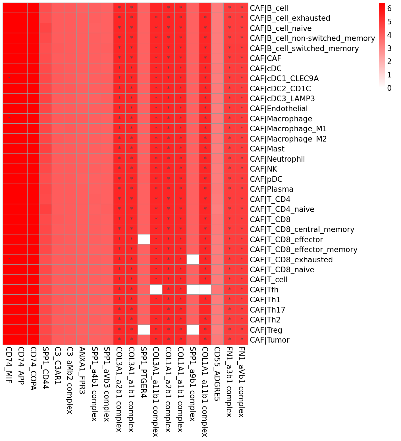

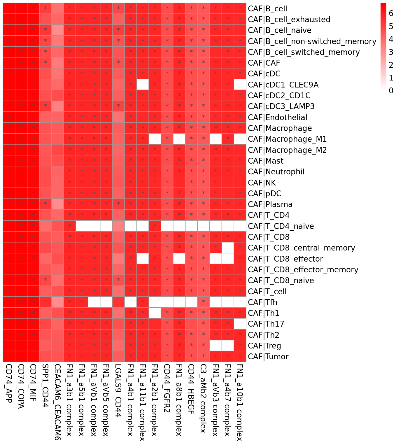
**

**P4 P5**

**
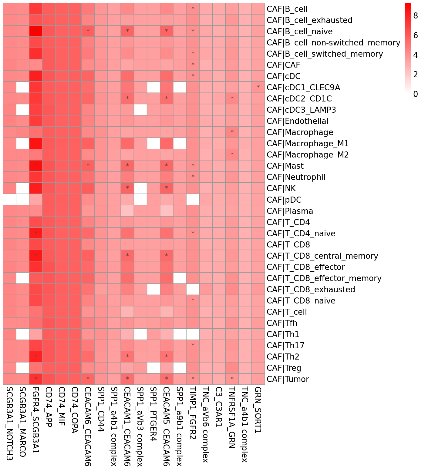

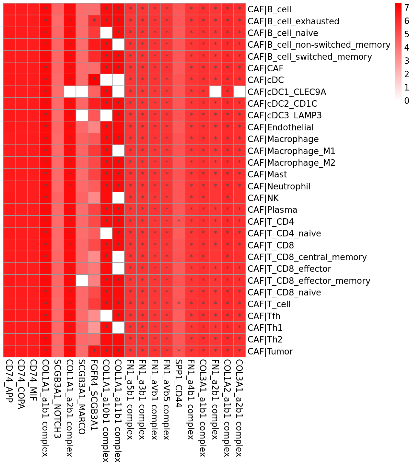
**
